# Plasma ceramides predict cardiovascular death in patients with stable coronary artery disease and acute coronary syndromes beyond LDL-cholesterol

**DOI:** 10.1093/eurheartj/ehw148

**Published:** 2016-04-28

**Authors:** Reijo Laaksonen, Kim Ekroos, Marko Sysi-Aho, Mika Hilvo, Terhi Vihervaara, Dimple Kauhanen, Matti Suoniemi, Reini Hurme, Winfried März, Hubert Scharnagl, Tatjana Stojakovic, Efthymia Vlachopoulou, Marja-Liisa Lokki, Markku S. Nieminen, Roland Klingenberg, Christian M. Matter, Thorsten Hornemann, Peter Jüni, Nicolas Rodondi, Lorenz Räber, Stephan Windecker, Baris Gencer, Eva Ringdal Pedersen, Grethe S. Tell, Ottar Nygård, Francois Mach, Juha Sinisalo, Thomas F. Lüscher

**Affiliations:** 1 Zora Biosciences, Espoo, Finland; 2 Medical School, Tampere University, Tampere, Finland; 3 Finnish Clinical Biobank Tampere, University Hospital of Tampere, Tampere, Finland; 4 Medical Clinic V (Nephrology, Hypertensiology, Rheumatology, Endocrinology, Diabetology), Medical Faculty Mannheim, University of Heidelberg, Heidelberg, Germany; 5 synlab Academy, synlab Holding Deutschland GmbH, Mannheim and Augsburg, Germany; 6 Clinical Institute of Medical and Chemical Laboratory Diagnostics, Medical University Graz, Graz, Austria; 7 Transplantation Laboratory, Haartman Institute, University of Helsinki, Helsinki, Finland; 8 Heart and Lung Center, Helsinki University Hospital, Helsinki, Finland; 9 Department of Cardiology, University Heart Center, University Hospital Zürich and University of Zürich, Zürich, Switzerland; 10 Institute of Clinical Chemistry, University Hospital, Zürich, Switzerland; 11 Applied Health Research Centre (AHRC), Li Ka Shing Knowledge Institute of St. Michael's Hospital, and Department of Medicine, University of Toronto, Toronto, Canada; 12 Department of General Internal Medicine, University Hospital Bern, Bern, Switzerland; 13 Department of Ambulatory Care and Community Medicine, University of Lausanne, Lausanne, Switzerland; 14 Cardiovascular Center, Department of Cardiology, University Hospital Bern, Bern, Switzerland; 15 Cardiovascular Center, Department of Cardiology, University Hospital Geneva, Geneva, Switzerland; 16 Department of Clinical Science, University of Bergen, Bergen, Norway; 17 Department of Global Public Health and Primary Care, University of Bergen, Bergen, Norway; 18 Department of Heart Disease, Haukeland University Hospital, Bergen, Norway

**Keywords:** Ceramide, Acute coronary syndrome, Coronary artery disease, Biomarker, LDL-cholesterol, Risk prediction, Prognosis

## Abstract

**Aims:**

The aim was to study the prognostic value of plasma ceramides (Cer) as cardiovascular death (CV death) markers in three independent coronary artery disease (CAD) cohorts.

**Methods and results:**

Corogene study is a prospective Finnish cohort including stable CAD patients (*n* = 160). Multiple lipid biomarkers and C-reactive protein were measured in addition to plasma Cer(d18:1/16:0), Cer(d18:1/18:0), Cer(d18:1/24:0), and Cer(d18:1/24:1). Subsequently, the association between high-risk ceramides and CV mortality was investigated in the prospective Special Program University Medicine—Inflammation in Acute Coronary Syndromes (SPUM-ACS) cohort (*n* = 1637), conducted in four Swiss university hospitals. Finally, the results were validated in Bergen Coronary Angiography Cohort (BECAC), a prospective Norwegian cohort study of stable CAD patients. Ceramides, especially when used in ratios, were significantly associated with CV death in all studies, independent of other lipid markers and C-reactive protein. Adjusted odds ratios per standard deviation for the Cer(d18:1/16:0)/Cer(d18:1/24:0) ratio were 4.49 (95% CI, 2.24–8.98), 1.64 (1.29–2.08), and 1.77 (1.41–2.23) in the Corogene, SPUM-ACS, and BECAC studies, respectively. The Cer(d18:1/16:0)/Cer(d18:1/24:0) ratio improved the predictive value of the GRACE score (net reclassification improvement, NRI = 0.17 and ΔAUC = 0.09) in ACS and the predictive value of the Marschner score in stable CAD (NRI = 0.15 and ΔAUC = 0.02).

**Conclusions:**

Distinct plasma ceramide ratios are significant predictors of CV death both in patients with stable CAD and ACS, over and above currently used lipid markers. This may improve the identification of high-risk patients in need of more aggressive therapeutic interventions.

## Introduction

Given the high prevalence of coronary artery disease (CAD) and associated mortality, prevention of fatal and non-fatal myocardial infarctions (MI) in CAD patients remains an ongoing clinical challenge. Mortality rates among stable CAD patients range between 1% and 3%, while rates of non-fatal events are 1–2% annually.^1^ In patients with acute coronary syndromes (ACS) who survive the acute event, the rate of MI and death is markedly higher, particularly during the first year.^2^ However, at the individual level, the event risk may vary considerably, which makes risk estimation tools necessary to improve patient management. Expedient risk stratification should identify individuals at risk requiring more intensive therapy. Conversely, patients with a favorable prognosis should be identified to avoid drug overuse and associated side effects.^3^

Hypothesis free lipidomic analyses have revealed a handful of lipids potentially qualifying as useful prognostic markers for CAD.^4–6^ In our initial lipidomic study, distinct ceramide species were significantly associated with CVD among CAD patients.^4^ Molecular lipid species, particularly ceramide(d18:1/16:0), were also associated with necrotic core tissue type and lipid core burden in coronary angiography, and were predictive for 1-year clinical outcome in 581 ACS and stable CAD patients.^7^ In these studies, plasma CVD risk-related ceramide molecules (Cer(d18:1/16:0), Cer(d18:1/18:0), and Cer(d18:1/24:1)), and their ratios with Cer(d18:1/24:0), emerged as potential risk stratifiers for CAD patients.^4^ Ceramides are known to associate with many central processes of atherosclerosis development including lipoprotein uptake, inflammation, and apoptosis (Supplementary material online, *Figure S1*).^8^ Ceramide species are produced by six fatty acyl selective ceramide synthases (CerSs; Supplementary material online, *Figure S2*), and it is becoming evident that individual ceramide species have specific physiological functions.^9–12^ Thus, monitoring ratios of ceramides species may provide insight into the metabolic regulation of atherosclerotic events. In this study, we establish the suggested role of ceramides and their distinct ratios as risk predictors for CV death in patients with stable CAD and ACS.

## Methods

More detailed method descriptions are available in Supplementary material online.

### Study subjects

#### Corogene study: stable coronary artery disease patients

Corogene is a prospective, consecutive cohort study of Finnish patients referred for coronary angiography to the Helsinki University Central Hospital between 2006 and 2008. A nested case control study was designed using the Corogene database and including data from the national death certificate registry. As cases, all patients who experienced coronary death within an average follow-up of 2½ years were selected. Matched control patients had established CAD (>50% stenosis at least in one epicardial coronary artery), but remained alive during the follow-up period. Baseline characteristics of the Corogene subjects are shown in *Table 1* and Supplementary material online, *Table S1*.

**Table 1:** Baseline characteristics of the subjects in Corogene, SPUM-ACS and BECAC studies

Characteristic	COROGENE		SPUM-ACS		BECAC	
	Cases	Controls	Cases	Controls	Cases	Controls
No of subjects	80	80	51	1586	81	1506
Gender						
Male, *n* (%)	60 (75%)	60 (75%)	42 (82%)	1223 (77%)	55 (68%)	889 (59%)
Age (years)	70.2 (62.6–77.1)	70 (63.4–76.9)	77.1 (69–83)	62.8 (53.9–72.9)	71 (64–78)	61 (54–70)
Body mass index	27.5 (23.6–30.9)	26 (24.1–29.5)	25.1 (23.1–28.3)	26.6 (24.3–29.4)	24 (22–28)	26 (23–28)
Time to death/follow-up time (days)	528 (134–739)	1955 (1669–2173)	25 (7–216)	365 (359–365)	626 (196–1332)	1720 (1368–2111)
Creatinine (µmol/L)	98 (84–134)	79 (69–90)	100 (79–134)	75 (65–88)	98 (87–115)	87 (79–97)
Current smoker						
Yes, *n* (%)	37 (46%)	37 (46%)	16 (31%)	663 (42%)	29 (36%)	355 (24%)
No, *n* (%)	43 (54%)	43 (54%)	33 (65%)	897 (57%)	50 (62%)	1146 (76%)
NA			2 (4%)	26 (2%)	2 (2%)	5 (0%)
Diabetes						
Yes, *n* (%)	32 (40%)	32 (40%)	11 (22%)	266 (17%)	16 (20%)	159 (11%)
No, *n* (%)	48 (60%)	48 (60%)	40 (78%)	1320 (83%)	64 (79%)	1333 (89%)
NA					1 (1%)	14 (1%)
Hypertension						
Yes, *n* (%)	60 (75%)	60 (75%)	36 (71%)	912 (58%)	55 (68%)	674 (45%)
No, *n* (%)	20 (25%)	20 (25%)	15 (29%)	674 (42%)	26 (32%)	832 (55%)
Lipid-lowering treatment						
Yes, *n* (%)	61 (76%)	61 (76%)	14 (27%)	432 (27%)	61 (75%)	933 (62%)
No, *n* (%)	19 (24%)	19 (24%)	34 (67%)	1146 (72%)	20 (25%)	573 (38%)
NA			3 (6%)			
Previous AMI						
Yes, *n* (%)	67 (84%)	0 (0%)	8 (16%)	210 (13%)	54 (67%)	482 (32%)
No, *n* (%)	13 (16%)	80 (100%)	43 (84%)	1374 (87%)	27 (33%)	1024 (68%)
NA				2 (0%)		
Previous stroke						
Yes, *n* (%)	17 (21%)	10 (12%)	2 (4%)	37 (2%)	15 (19%)	108 (7%)
No, *n* (%)	63 (79%)	70 (88%)	49 (96%)	1549 (98%)	66 (81%)	1398 (93%)

#### Bergen Coronary Angiography Cohort cohort: patients with stable coronary artery disease

The Bergen Coronary Angiography Cohort (BECAC) includes 1580 adults referred to elective coronary angiography because of suspected stable angina pectoris recruited at the Haukeland University Hospital in Bergen, Norway between 2000 and 2004. Information on cardiovascular deaths was collected from the Cause of Death Registry at the Norwegian Institute of Public Health, and verified against hospital medical records whenever available. During a median follow-up of 4.6 years, a total of 81 patients died from cardiovascular disease. Baseline characteristics of the BECAC participants are reported in *Table 1* and Supplementary material online, *Table S1*.

#### SPUM-ACS cohort: patients with acute coronary syndromes

Special Program University Medicine—Inflammation in Acute Coronary Syndromes (SPUM-ACS) is a prospective, multi-centre (Bern, Geneva, Lausanne, and Zürich) cohort study. Patients with a primary diagnosis of ACS and referred for invasive management were enrolled at four Swiss university hospitals. Baseline characteristics of the SPUM-ACS patients are summarized in *Table 1* and Supplementary material online, *Table S1*. At one-year follow-up, a total of 51 patients died from cardiac reasons.

### Clinical laboratory analyses

Standard lipids measurements were determined using standard methods available at each of the three study sites. In Corogene subjects, apolipoproteins (AI, AII, and B), lipoprotein (a), lipoprotein-associated phospholipase A2 activity, and HDL and LDL particle numbers and sizes were measured as described in Supplementary material online, Methods.

### Quantification of ceramides

The plasma levels of Cer(d18:1/16:0), Cer(d18:1/18:0), Cer(d18:1/24:0), and Cer(d18:1/24:1) were quantified on a 5500 QTRAP (SCIEX, Framingham, MA) mass spectrometer equipped with an Eksigent 100-XL UHPLC system as described recently.^13^

### Statistical analyses

Wilcoxon's rank sum test was applied for group comparisons. Odds ratios (ORs) per standard deviation were estimated using logistic regression. Hazard ratios were calculated using the Cox proportional hazard model. The GRACE^14^ risk score, consisting of Killip class, systolic blood pressure, heart rate, age, creatinine, cardiac arrest at admission, ST-segment deviation, and elevated cardiac enzyme levels (troponin, CK-MB), was used to calculate the risk of long-term mortality for ACS patients. The following Marschner score^15^ variables were used in the modeling of stable CAD patient data: total cholesterol, HDL-C, age, gender, smoking status, previous acute MI, diabetes, hypertension, and prior stroke.

Net reclassification improvement was estimated as described by Pencina *et al.*^16^ For the 1-year event risk of the secondary prevention population in the SPUM-ACS study we categorized subjects to low risk (<1% event probability), intermediate (1–5%) risk or high-risk (>5%) groups. For the BECAC study the same categorization was used for 3-year risk.

The ceramide risk score was calculated as follows: For each individual, all three ceramide ratios and each concentration (apart from Cer(d18:1/24:0)) were compared with the whole study population. If the variable belonged to the 3rd quartile, the individual received +1 point, and if to the 4th quartile, +2 points (Supplementary material online, *Table S11*). Thus, the score ranges from 0 to 12 and based on the score, the subjects were split into four risk categories (0–2, 3–6, 7–9, and 10–12).

More details on statistical methods can be found in Supplementary material online.

## Results

### Ceramide concentrations in high- and low-risk patients with coronary artery disease

In stable CAD patients of the Corogene study LDL-based markers such as LDL-C, LDL particle number (LDL-P), small dense LDL (sdLDL), and apoB did not differ significantly between cases who experienced coronary death and controls who remained alive, and neither did Lp(a) nor Lp-PLA2. However, the HDL-related markers HDL-C, HDL particle number (HDL-P), small dense HDL (sdHDL), and ApoA1 were all significantly (*P* < 0.001) different between the groups with the medians being −17.1, −14.3, −19.0, and −12.9% lower in cases, respectively. The differences between cases and controls in plasma ceramides and established lipid markers are provided in *Table 2* (the percentage of observations for each marker is provided in Supplementary material online, *Table S2*).

**Table 2:** Medians and inter quartile ranges of established lipid markers and ceramides in case and control groups^a^

	BECAC			SPUM-ACS		
	Cases (*n* = 81)	Controls (*n* = 1499)	*P*-value	Cases (*n* = 51)	Controls (*n* = 1586)	*P*-value
Cer(d18:1/16:0)/Cer(d18:1/24:0)	0.121 (0.101–0.145)	0.100 (0.085–0.119)	<0.001	0.116 (0.099–0.170)	0.093 (0.079–0.113)	<0.001
Cer(d18:1/18:0)/Cer(d18:1/24:0)	0.046 (0.036–0.059)	0.038 (0.031–0.049)	<0.001	0.064 (0.044–0.084)	0.047 (0.037–0.060)	<0.001
Cer(d18:1/24:1)/Cer(d18:1/24:0)	0.498 (0.408–0.624)	0.413 (0.337–0.508)	<0.001	0.489 (0.415–0.675)	0.394 (0.337–0.474)	<0.001
Cer(d18:1/16:0) (µmol/L)	0.271 (0.235–0.326)	0.253 (0.213–0.300)	0.010	0.313 (0.255–0.385)	0.292 (0.247–0.346)	0.090
Cer(d18:1/18:0) (µmol/L)	0.108 (0.077–0.143)	0.096 (0.076–0.123)	0.097	0.161 (0.109–0.234)	0.146 (0.112–0.189)	0.163
Cer(d18:1/24:0) (µmol/L)	2.335 (1.843–2.866)	2.548 (2.030–3.098)	0.035	2.366 (2.112–3.084)	3.107 (2.490–3.826)	<0.001
Cer(d18:1/24:1) (µmol/L)	1.056 (0.927–1.344)	1.028 (0.844–1.257)	0.026	1.421 (1.012–1.628)	1.229 (1.004–1.484)	0.175
LDL-C (mg/dL)	110 (89–133)	116 (93–147)	0.087	101 (81–128)	121 (93–150)	0.001
HDL-C (mg/dL)	46 (35–58)	50 (41–62)	0.036	48 (36–58)	44 (36–53)	0.266
TC (mg/dL)	185 (158–212)	193 (166–224)	0.081	159 (147–189)	189 (161–221)	<0.001
TG (mg/dL)	135 (100–169)	126 (92–182)	0.738	76 (54–108)	92 (61–142)	0.014
	**COROGENE**					
	**Cases (*n* = 80)**	**Controls (*n* = 80)**	***P*-value**			
Cer(d18:1/16:0)/Cer(d18:1/24:0)	0.132 (0.105–0.175)	0.105 (0.090–0.128)	<0.001			
Cer(d18:1/18:0)/Cer(d18:1/24:0)	0.062 (0.047–0.077)	0.046 (0.037–0.062)	<0.001			
Cer(d18:1/24:1)/Cer(d18:1/24:0)	0.703 (0.582–0.846)	0.556 (0.483–0.665)	<0.001			
Cer(d18:1/16:0) (µmol/L)	0.275 (0.222–0.326)	0.235 (0.212–0.282)	0.007			
Cer(d18:1/18:0) (µmol/L)	0.118 (0.094–0.152)	0.107 (0.092–0.137)	0.195			
Cer(d18:1/24:0) (µmol/L)	1.923 (1.475–2.511)	2.235 (1.993–2.672)	0.008			
Cer(d18:1/24:1) (µmol/L)	1.385 (1.189–1.620)	1.245 (1.091–1.427)	0.017			
TC (mg/dL)	128 (111–165)	139 (122–163)	0.064			
TG (mg/dL)	108 (86–140)	92 (75–139)	0.110			
LDL-C (mg/dL)	69 (55–99)	75 (65–92)	0.251			
LDL-P (nmol/L)	830 (694–1110)	928 (712–1175)	0.395			
sdLDL (nmol/L)	533 (304–659)	548 (376–737)	0.265			
ApoB (mg/dL)	67 (55–82)	68.5 (57–84)	0.997			
HDL-C (mg/dL)	34 (29–40)	41 (33–51)	<0.001			
HDL-P (µmol/L)	24 (21–27)	28 (24–31)	<0.001			
sdHDL (µmol/L)	12.8 (9.3–15.6)	15.8 (13.1–18.2)	<0.001			
ApoA1 (mg/dL)	115 (101–131)	132 (115–150)	<0.001			
Lp(a) (mg/dL)	7.2 (2–35)	3.6 (1–28)	0.319			
Lp-PLA2 (nmol/min/ml)	138 (119–166)	130 (115–163)	0.354			
C-reactive protein (mg/L)	3.1 (1.6–8.7)	1.1 (0.7–2.8)	<0.001			

^a^Cer, ceramide; TC, total cholesterol; TG, triacylglycerols, LDL-C low-density lipoprotein cholesterol, HDL-C high-density lipoprotein cholesterol, sdLDL small dense low-density lipoprotein cholesterol, LDL-P low-density lipoprotein particle number, sdHDL small dense high-density lipoprotein cholesterol, HDL-P high-density lipoprotein particle number, ApoB apolipoprotein B, ApoA1 apolipoprotein A1, Lp(a) lipoprotein (a), Lp-PLA2 lipoprotein-associated phospholipase A2. SI conversion factors: To convert cholesterol to mmol/L, multiply values by 0.0259; to convert triacylglycerols to mmol/L, multiply values by 0.01129.

In the Corogene study, the concentrations of Cer(d18:1/16:0), Cer(d18:1/18:0), and Cer(d18:1/24:1) were significantly different (*P* < 0.001 for all) between cases who had a fatal MI during the follow-up period and controls (medians in cases +17.0%, +10.3, and +11.2% higher than in controls, respectively). In contrast, the Cer(d18:1/24:0) behaved differently, with the median in cases being −14.9% lower than in controls (*P* < 0.001). Similarly to our earlier observations,^4^ highly significant differences, were observed for the three predefined ceramide ratios, with the medians of cases ranging between +25.7 and +34.8% (*P* < 0.001) relative to controls. The difference between stable CAD patients and controls is illustrated in Supplementary material online, *Figure S3*.

### Ceramide ratios in coronary artery disease patients

In the Corogene study, the most significant ORs for coronary death were found for HDL markers and ceramide ratios. Ceramide ORs remained predictive after adjustment for conventional lipid markers LDL-C, HDL-C, total cholesterol, triacylglycerols, and C-reactive protein. Lp-PLA2 and Lp(a) had no significant association with CV mortality.

**Figure 1:**
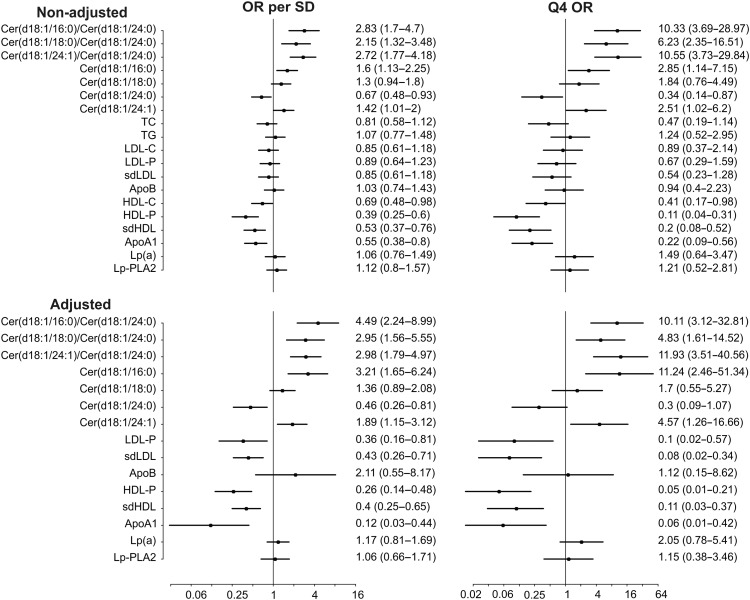
Cardiovascular death odds ratios for per standard deviation and 4th quartile for different lipid markers and ceramides in Corogene study. Adjustment is made for total cholesterol, triacylglycerols, LDL-C, HDL-C, and C-reactive protein.

LDL-C and LDL particle number were inversely associated with risk (LDL-C unadjusted upper quartile OR 0.89 95% CI 0.37–2.14; LDL-P upper quartile OR 0.67, 95%CI 0.29–1.59). For comparison, the unadjusted upper quartile OR for the Cer(d18:1/16:0)/Cer(d18:1/24:0) ratio was 10.33 (95% CI 3.69–28.97). *Figure 1* shows non-adjusted and adjusted ORs for different lipid markers and ceramides in the Corogene study.

### Ceramides and risk in stable coronary artery disease patients

An independent assessment of ceramides was performed in a cohort (BECAC) of stable patients. We found that the predefined ceramide ratios were significantly higher in 81 patients who died following a CV event within 4.6-year follow-up compared with those who did not die during follow-up (*Table 2*; Supplementary material online, *Table S2*). For comparability with the Corogene results, non-adjusted and adjusted ORs for standard lipid markers and ceramides are given in Supplementary material online, *Table S3*. The incremental improvement of discrimination for CV death was further demonstrated by calculating hazard ratios adjusted for standard lipids and the Marschner score variables (*Table 3*).

**Table 3:** Association between ceramides and cardiovascular death in BECAC^a^

	Univariate model		Multivariable^b^ model 1^b^		Multivariable^c^ model 2^c^	
	Hazard ratio^d^ (95% CI)	*P*-value	Hazard ratio^d^ (95% CI)	*P*-value	Hazard ratio^d^ (95% CI)	*P*-value
Cer(d18:1/16:0)/Cer(d18:1/24:0)^e^	1.77 (1.46–2.16)	<0.001	1.79 (1.45–2.20)	<0.001	1.52 (1.21–1.92)	<0.001
Cer(d18:1/18:0)/Cer(d18:1/24:0)^e^	1.63 (1.31–2.04)	<0.001	1.58 (1.25–2.00)	<0.001	1.29 (1.01–1.65)	0.039
Cer(d18:1/24:1)/Cer(d18:1/24:0)^e^	1.61 (1.30–1.98)	<0.001	1.58 (1.27–1.97)	<0.001	1.31 (1.03–1.66)	0.028
Cer(d18:1/16:0)^e^	1.44 (1.17–1.77)	<0.001	2.09 (1.61–2.73)	<0.001	1.75 (1.30–2.35)	<0.001
Cer(d18:1/18:0)^e^	1.33 (1.07–1.65)	0.011	1.54 (1.19–2.01)	0.001	1.27 (0.98–1.66)	0.076
Cer(d18:1/24:0)^e^	0.83 (0.67–1.02)	0.081	0.82 (0.62–1.10)	0.182	0.91 (0.69–1.21)	0.510
Cer(d18:1/24:1)^e^	1.39 (1.13–1.72)	0.002	1.74 (1.34–2.25)	<0.001	1.38 (1.04–1.82)	0.023

Cer denotes ceramide.

^a^CV death denotes death from MI, stroke, and heart failure.

^b^The model was adjusted for TC, TG, HDL-C, and LDL-C.

^c^The model was adjusted as for model 1 with additional adjustment for the following Marschner score variables: age, gender, smoking status, previous acute MI, diabetes, hypertension, and prior stroke.

^d^Hazard ratios are for 1 SD increase.

^e^Natural logarithm of the ceramides and ceramide ratio.

Adjustment for statin treatment did not have a major impact on the results (Supplementary material online, *Table S4*). The odds ratios were calculated for ceramides and LDL-C also in patients that were on or not on statin treatment both at baseline and after 1-year of follow-up (Supplementary material online, *Table S5*). Ceramides were predictive in both instances, although in patients without statin treatment the odds ratios were better. LDL-C did not show significant predictive value, confirming that the lack of a direct association between LDL-C and CV death was not caused by interference with statin treatment.

The incremental prognostic value of ceramides was tested by comparing the base model composed of the Marschner score variables to a new model with the Marschner score variables combined with the Cer(d18:1/16:0)/Cer(d18:1/24:0) ratio. The ceramides increased the cross-validated c-statistics from 0.78 (0.75–0.80) to 0.80 (0.77–0.82). Further, the predicted probabilities for a 1-year event risk by logistic regression yielded an NRI of 0.15 (95% CI 0.06–0.25; 9.6% improvement for events and 5.8% improvement for non-events).

### Ceramides and risk prediction in acute coronary syndromes patients

Another independent assessment of ceramides was performed in the SPUM-ACS cohort enrolling ACS patients. In 51 patients who died following a cardiac event within one-year-follow-up the ceramide ratios were significantly higher compared with those who survived during follow-up (*Table 2*; Supplementary material online, *Table S2*). For comparability with the Corogene results, non-adjusted and adjusted ORs for standard lipid markers and ceramides are given in Supplementary material online, *Table S3*, and the effects of statin treatment are accounted for the results in Supplementary material online, *Table S4*. The incremental improvement of discrimination for cardiac death was further demonstrated by adjusting for standard lipids and the GRACE score (*Table 4*), and also by taking into account diabetes mellitus and smoking status (Supplementary material online, *Table S6*).

**Table 4:** Association between ceramides and cardiovascular death in SPUM-ACS^a^

	Univariate model		Multivariable^b^ model 1^b^		Multivariable^c^ model 2^c^	
	Hazard ratio^d^ (95% CI)	*P*-value	Hazard ratio^d^ (95% CI)	*P*-value	Hazard ratio^d^ (95% CI)	*P*-value
Cer(d18:1/16:0)/Cer(d18:1/24:0)	1.81 (1.52–2.14)	<0.001	1.82 (1.51–2.21)	<0.001	1.69 (1.39–2.06)	<0.001
Cer(d18:1/18:0)/Cer(d18:1/24:0)	1.66 (1.43–1.96)	<0.001	1.65 (1.39–1.97)	<0.001	1.48 (1.24–1.76)	<0.001
Cer(d18:1/24:1)/Cer(d18:1/24:0)	1.74 (1.45–2.08)	<0.001	1.77 (1.44–2.17)	<0.001	1.64 (1.32–2.03)	<0.001
Cer(d18:1/16:0)^e^	1.45 (1.10–1.93)	0.010	1.96 (1.45–2.66)	<0.001	1.98 (1.49–2.62)	<0.001
Cer(d18:1/18:0)^e^	1.43 (1.07–1.90)	0.015	1.77 (1.31–2.38)	<0.001	1.66 (1.26–2.20)	<0.001
Cer(d18:1/24:0)^e^	0.66 (0.51–0.87)	0.003	0.74 (0.52–1.05)	0.090	0.91 (0.65–1.29)	0.609
Cer(d18:1/24:1)^e^	1.23 (0.93–1.63)	0.154	1.74 (1.25–2.42)	0.001	1.73 (1.27–2.36)	<0.001

Cer denotes ceramide.

^a^CV death denotes death from MI, stroke, and heart failure.

^b^The model was adjusted for TC, TG, HDL-C, and LDL-C.

^c^The model was adjusted as for model 1 with additional adjustment for the Grace score (Killip class, systolic blood pressure, heart rate, age, creatinine, cardiac arrest at admission, ST-segment deviation, and elevated cardiac enzyme levels).

^d^Hazard ratios are for one standard deviation increase.

^e^Natural logarithm of the ceramides.

The incremental prognostic value of ceramides was tested by comparing the base model composed of the GRACE score to a new model with the GRACE score and the Cer(d18:1/16:0)/Cer(d18:1/24:0) ratio on top. The ceramide ratio increased the cross-validated c-statistics from 0.73 (0.70–0.77) to 0.82 (0.79–0.85). Furthermore, the predicted probabilities for a 1-year event risk obtained by logistic regression yielded an NRI of 0.17 (95% CI 0.07–0.27; 8.2% improvement for events and 9.1% improvement for non-events).

The performance of the ceramide ratio Cer(d18:1/16:0)/Cer(d18:1/24:0) in predicting non-fatal MI was also investigated by calculating the hazard ratios both for Q-wave and non-Q wave MIs. The ratio showed a significant result for Q-wave MI, while no significant results were obtained for non-Q wave infarctions (Supplementary material online, *Table S7*).

### Ceramides and C-reactive protein

In the Corogene and SPUM-ACS studies, ceramides associated significantly with LDL-C and C-reactive protein. Particularly, the CV mortality-related Cer(d18:1/16:0) and Cer(d18:1/18:0) were positively correlated with C-reactive protein, while small negative correlations were seen in both studies between C-reactive protein and Cer(d18:1/24:0). Furthermore, the ‘protective’ Cer(d18:1/24:0) had the strongest associations with LDL-C. Correlation coefficients for associations of ceramides and ceramide ratios with LDL-C and C-reactive protein are presented in Supplementary material online, *Tables S8* and *S9*.

Finally, the synergy of C-reactive protein and Cer(d18:1/16:0)/Cer(d18:1/24:0) in risk stratification was investigated by calculating event rates in different quartiles for both BECAC and SPUM-ACS. Especially in the SPUM-ACS study the highest enrichment of events (11.4% 1-year mortality) was observed if both the ceramide ratio and C-reactive protein were in the highest quartile of the whole population (Supplementary material online, *Table S10*).

### Ceramide score and risk for cardiovascular death

We have developed a tentative risk score (Supplementary material online, *Table S11*) based on ceramide concentrations and their ratios to model the clinical use of these risk predictors. Based on the score, the patients were placed into four risk categories (low–moderate–increased–high) and both in the BECAC and SPUM-ACS studies the risk increased along with the increasing score (*Table 5*). In the stable CAD and ACS patients 4.2- and 6.0-fold relative risk increase was observed when comparing the high- to low-risk category, respectively. When subjects were sorted according to their LDL-C concentrations and split into four categories in the same proportion as for the ceramide risk score the enrichment of high-risk patients was not observed along with increasing LDL-C concentration.

**Table 5:** Ceramide score and risk for cardiovascular death

BECAC (5-year risk)					SPUM-ACS (1-year risk)				
Score	No death	Death	%	Relative risk	Score	No death	Death	%	Relative risk
0–2	534	15	2.7%	1.0	0–2	566	9	1.6%	1.0
3–6	572	29	4.8%	1.8	3–6	595	16	2.6%	1.7
7–9	268	20	6.9%	2.5	7–9	261	9	3.3%	2.1
10–12	132	17	11.4%	4.2	10–12	164	17	9.4%	6.0
**LDL-C (mg/dl)**	**No death**	**Death**	**%**	**Relative risk**	**LDL-C (mg/dl)**	**No death**	**Death**	**%**	**Relative risk**
≤100	513	36	6.6%	1.0	≤106	532	27	4.8%	1.0
100–143	572	29	4.8%	0.7	106–145	576	17	2.9%	0.6
143–175	278	10	3.5%	0.5	145–174	260	3	1.1%	0.2
≥175	142	6	4.1%	0.6	≥174	174	2	1.1%	0.2

See Supplementary material online, *Table S11* for information on Ceramide Score calculation. To compare Ceramide Score performance with that of LDL-C study, subjects were sorted according to their LDL-C levels and split into four categories in the same proportion as for the ceramide risk score.

## Discussion

The present results provide evidence that distinct ceramide species serve as significant predictors for cardiovascular death beyond currently used lipid markers in two patient groups—patients with stable CAD and higher risk ACS patients . Importantly, the prediction also works in patients who are already statin treated and is therefore a potential indicator of residual risk.

Battes *et al.*^17^ recently performed a systematic review of models predicting outcome in patients with stable CAD. The authors concluded that risk stratification should be improved to predict recurrent coronary events and to optimize secondary prevention strategies. Our data using ceramides address this unmet need and show robust performance for predicting coronary death both in stable CAD and ACS patients. The present results do not prove causality. However, it is tempting to speculate that ceramides are associated with plaque vulnerability as they are known to fuel many central atherosclerosis processes including lipoprotein aggregation and uptake, inflammation, superoxide anion production, and apoptosis^8,18–21^ (Supplementary material online, *Figure S1*). Several enzymes of the sphingolipid synthesis have already been tested as potential drug targets as inhibition of glycosphingolipid biosynthesis has been shown to decrease atherosclerosis in mice.^10,22^ Evidence is also accumulating on ceramide chain-length-specific functions. In a recent study, the relative increase in long-chain species (C16) but not in very-long-chain (C24-24:1) species was shown to mediate insulin resistance in mice.^11,12^ In *Caenorhabditis elegans*, long-chain ceramides were pro-apoptotic, and very-long-chain ceramides were anti-apoptotic.^9^ Consistently in the present study, long-chain species (d18:1/16:0 and d18:1/18:0) were more harmful than very-long-chain (d18:1/24:0) species. Altered ceramide compositions may partially be explained by CerS isoforms, providing a putative biological explanation for the use of ceramide ratios, and possibilities for medical intervention (Supplementary material online, *Figure S2*). Interestingly, Cer(d18:1/24:1) behaved differently compared with Cer(d18:1/24:0), emphasizing that additional regulation also takes place. While the current study reveals an association between ceramides and CV events, it will be a highly interesting topic for future investigations to establish if ceramide composition can be influenced and how it might translate to cardiovascular benefit. The response of ceramides to lipid-lowering treatments such as statins has been documented in our previous study.^4^ We have observed that PCSK9 knock-out mice have significantly reduced plasma ceramide concentrations and that human PCSK9 loss-of-function mutations are associated with lower plasma ceramide concentrations compared with individuals carrying the major alleles.^4,23^ Study limitations in addition to the lack of causality data include the limited number of events both in BECAC and SPUM-ACS. Thus, the ceramide risk score derived from these studies should be further validated in sizeable cohorts in order to fine-tune the relative risk estimates for different risk categories. Finally, it is likely that the careful one-to-one case–control matching in the Corogene study is leading to somewhat optimistic biomarker results compared with a real-life patient care situation where controlling for confounding factors is more difficult.

The lack of a discernible relationship between LDL-related parameters and CV risk across the studies included here, even after statin stratification, is thought-provoking but, in line with previous reports.^24–26^ For example, Sachdeva *et al*.^24^ analysed admission lipid levels in a broad population of 136,995 patients hospitalized for CAD in 541 hospitals and observed that nearly half of the admission LDL-C concentrations were <100 mg/dl although before admission only 21.1% patients were receiving lipid-lowering medications. Furthermore, in the MIRACL trial, the plasma HDL-C, but not LDL-C, measured in the initial stage of ACS predicted the risk of recurrent cardiovascular events.^25^ The lower LDL-C in higher risk patients may not be a phenomenon of ACS solely as in the Saturn trial investigators observed that C-reactive protein, but not LDL-C levels, were associated with coronary atheroma regression and cardiovascular events after intensive statin therapy.^26^ Taken together, it appears that the LDL-C concentrations may be similar or even lower in CAD patients at high risk for future CV events compared with patients with more favorable prognosis. This may be a phenomenon related to disease progression and culmination, and should not detract from the value of applying LDL-C to gauge the lifetime risk to develop atherosclerotic plaques. A potential explanation for this is provided in Gierens *et al.*^27^ who demonstrated that interleukin-6 (IL-6) activates LDL-receptor (LDLr) transcription and subsequently enhances LDLr activity in the liver leading to an increased elimination of LDL-C from the circulation. Thus, chronic, and in particular acute bursts of, inflammation in CAD patients may enhance LDL-C clearance, resulting in lowered blood LDL-C concentrations and impaired risk prediction.

Ceramide measurement in high-throughput quality controlled environments is straightforward and cost-efficient. Isotope labelled standards enable precise quantification and analytical stability. Most clinical laboratories are equipped with robotized sample handling systems and also house mass spectrometry equipment.

Thus, ceramide-based identification of coronary patients at high cardiovascular risk will soon be possible. These high-risk patients should then benefit from treatments that extend beyond standard care. The suggested actions could include more frequent follow-up visits and efficient life-style counselling as well as the consideration for higher statin doses, ezetimibe combinations, or novel therapies such as PCSK9 inhibitors. In future, additional therapies may include other options, for example, ongoing randomized clinical trials are looking into the effect of methotrexate and interleukin-1β inhibition for treating cardiovascular risk.^28^ Indeed, ceramides are closely linked to inflammatory processes and recently CERS6 has been identified as a target for methotrexate.^29^ It is hence plausible to think that ceramide testing could become increasingly relevant, especially if the trials with anti-inflammatory compounds turn out positive. A health economic dimension of ceramide testing is its ability to target more intense, potentially more expensive treatments such as PCSK9 inhibitors to those at the highest risk. Another aspect of ceramide testing is its potential for motivating patient's adherence, whether for medication or life-style changes, due to its rather direct linkage with CV mortality. It has been shown that over 40% of the patients prescribed statins are non-adherent, which may translate to many avoidable additional events and hospitalizations.^30^

While the ceramide-based risk stratification extends beyond the current lipid-based diagnostics and addresses the unmet need for improved identification of high-risk CAD patients there are further scientific and clinical issues that need attention. There are two major lines of future research concerning the present ceramide correlation and cardiovascular risk. On one hand it is of interest to pursue the biology of these molecules and work out their molecular mechanism of action in cardiovascular disease. This will involve multi-disciplinary efforts of cell biologists, biochemists, geneticists, and clinicians developing appropriate cell and animal models. Efforts in this regard are already being made for example in the scope of the EU funded ‘EUFP7-Atheroflux’ consortium. The other line to follow is to establish the utility of these markers in clinical practice. In the USA, the ceramide testing is entering the clinic this year and only this real-life evaluation will allow for a better judgement of the ceramide utility and will establish them as a new armament in the clinical diagnostic tool-kit.

## Supplementary material

Supplementary material is available at *European Heart Journal* online.

## Authors’ contributions

M. S.-A., M. H., M. S. performed statistical analysis; R. L., R. H., M. N., J. S., O. N., T. L., W. M. handled funding and supervision; K. E., D. K., H. S., T. S., E. V., M.-L. L., R. K., C. M., T. H., P. J., N. R., L. R., S. W., B. G., E. R. P., G. S. T., F. M. acquired the data; R. L., R. H., J. S., T. L., F. M., O. N. conceived and designed the research; R. L., T. V. drafted the manuscript; K. E., M. S.-A., M. H., R. H., D. K., W. M., H. S., T. S., E. V., M. L. L., M. N., R. K., C. M., T. H., P. J., N. R., L. R., S. W., E. P., G. T., B. G., F. M., J. S., O. N., T. L. made critical revision of the manuscript for key intellectual content.

## Funding

This work was supported by the European Union's Seventh Framework Programme FP7/2007-2013 RiskyCAD Project (305739^2^) and further by research grants of the Swiss National Research Foundation (SPUM 33CM30-124112), the Swiss Heart Foundation, both Bern Switzerland, the Foundation for Cardiovascular Research—Zürich Heart House, Zürich, Switzerland as well as AstraZeneca, Zug; Eli Lilly Indianapolis, USA; and Vernier, Medtronic, Tolochenaz; Merck Sharpe and Dohme, Glattbrugg; Sanofi, Vernier; and St. Jude Medical, Zürich (all Switzerland). The Corogene study was supported by grants from Aarno Koskelo Foundation, Helsinki University Central Hospital Special Government Funds (EVO #TYH7215, #TKK2012005, #TYH2012209, and #TYH2014312), and Finnish Foundation for Cardiovascular research. The BECAC study was supported by a grant from the Western Norway Regional Health Authority (911570). The funders had no role in the design and conduct of the study; collection, management, analysis, and interpretation of the data; preparation, review, or approval of the manuscript; and decision to submit the manuscript for publication. Funding to pay the Open Access publication charges for this article was provided by Zora Biosciences.

**Conflict of interest:** F.M. has received research grants to the institution from Amgen, AstraZeneca, Boston Scientific, Biotronik, Medtronic, MSD, Eli Lilly, and St. Jude Medical including speaker or consultant fees. S.W. has received research grants to the institution from Abbott, AstraZeneca, Boston Scientific, Biosensors, Biotronik, Cordis, Eli Lilly, Medtronic, and St. Jude Medical. T.F.L. received research grants to the institution from AstraZeneca, Bayer Health Care, Biosensors, Biotronik, Boston Scientific, Medtronic, Merck, Sharpe and Dohme, Merck, Inc., Roche, and Servier, including lecture fees. C.M.M. received research grants to the institution from Eli Lilly, AstraZeneca, Roche and MSD and speaker or consultant fees from Eli Lilly, Daiichi Sankyo, AstraZeneca, Roche and MSD. W.M. is employed with Synlab Holding Germany GmbH, has ownership interest in Synlab Holding International GmbH and has received research grants to the institution from Aegerion Pharmaceuticals, Amgen, Astrazeneca, Danone Research, Sanofi/Genzyme, Hoffmann LaRoche, Numares, Unilever, and BASF and speaker and consultancy fees from Aegerion Pharmaceuticals, Amgen, Astrazeneca, Danone Research, Sanofi/Genzyme, Hoffmann LaRoche, Merck Sharp and Dohme, Pfizer, Sanofi, Synageva, Numares, Unilever, and BASF. Zora Biosciences holds patents for the diagnostic use of ceramides and R.H. and R.L. are shareholders of Zora Biosciences.

## Supplementary Material

Supplementary Data

## References

[1] Morrow DA Cardiovascular risk prediction in patients with stable and unstable coronary heart disease. Circulation 2010;121:2681–2691. 20566966 10.1161/CIRCULATIONAHA.109.852749

[2] Hamm CW Bassand J-P Agewall S Bax J Boersma E Bueno H Caso P Dudek D Gielen S Huber K Ohman M Petrie MC Sonntag F Uva MS Storey RF Wijns W Zahger D ESC Guidelines for the management of acute coronary syndromes in patients presenting without persistent ST-segment elevation: The Task Force for the management of acute coronary syndromes (ACS) in patients presenting without persistent ST-segment elevatio. Eur Heart J 2011;32:2999–3054. 21873419 10.1093/eurheartj/ehr236

[3] Montalescot G Sechtem U Achenbach S Andreotti F Arden C Budaj A Bugiardini R Crea F Cuisset T Di Mario C Ferreira JR Gersh BJ Gitt AK Hulot J-S Marx N Opie LH Pfisterer M Prescott E Ruschitzka F Sabaté M Senior R Taggart DP van der Wall EE Vrints CJM Zamorano JL Baumgartner H Bax JJ Bueno H Dean V Deaton C Erol C Fagard R Ferrari R Hasdai D Hoes AW Kirchhof P Knuuti J Kolh P Lancellotti P Linhart A Nihoyannopoulos P Piepoli MF Ponikowski P Sirnes PA Tamargo JL Tendera M Torbicki A Wijns W Windecker S Valgimigli M Claeys MJ Donner-Banzhoff N Frank H Funck-Brentano C Gaemperli O Gonzalez-Juanatey JR Hamilos M Husted S James SK Kervinen K Kristensen SD Pietro MA Pries AR Romeo F Rydén L Simoons ML Steg PG Timmis A Yildirir A 2013 ESC guidelines on the management of stable coronary artery disease: the Task Force on the management of stable coronary artery disease of the European Society of Cardiology. Eur Heart J 2013;34:2949–3003. 23996286 10.1093/eurheartj/eht296

[4] Tarasov K Ekroos K Suoniemi M Kauhanen D Sylvänne T Hurme R Gouni-Berthold I Berthold HK Kleber ME Laaksonen R März W Molecular lipids identify cardiovascular risk and are efficiently lowered by simvastatin and PCSK9 deficiency. J Clin Endocrinol Metab 2014;99:E45–E52. 24243630 10.1210/jc.2013-2559 PMC3928964

[5] Meikle PJ Wong G Tsorotes D Barlow CK Weir JM Christopher MJ MacIntosh GL Goudey B Stern L Kowalczyk A Haviv I White AJ Dart AM Duffy SJ Jennings GL Kingwell BA Weir M Plasma lipidomic analysis of stable and unstable coronary artery disease. Arterioscler Thromb Vasc Biol 2011;31:2723–2732. 21903946 10.1161/ATVBAHA.111.234096

[6] Fernandez C Sandin M Sampaio JL Almgren P Narkiewicz K Hoffmann M Hedner T Wahlstrand B Simons K Shevchenko A James P Melander O Plasma lipid composition and risk of developing cardiovascular disease. PLoS ONE 2013;8:e71846. 23967253 10.1371/journal.pone.0071846 PMC3744469

[7] Cheng JM Garcia-Garcia HM de Boer SPM Kardys I Heo JH Akkerhuis KM Oemrawsingh RM van Domburg RT Ligthart J Witberg KT Regar E Serruys PW van Geuns R-J Boersma E In vivo detection of high-risk coronary plaques by radiofrequency intravascular ultrasound and cardiovascular outcome: results of the ATHEROREMO-IVUS study. Eur Heart J 2014;35:639–647. 24255128 10.1093/eurheartj/eht484

[8] Bismuth J Lin P Yao Q Chen C Ceramide: a common pathway for atherosclerosis? Atherosclerosis 2008;196:497–504. 17963772 10.1016/j.atherosclerosis.2007.09.018 PMC2924671

[9] Menuz V Howell KS Gentina S Epstein S Riezman I Fornallaz-mulhauser M Hengartner MO Gomez M Riezman H Martinou J Protection of C. elegans from Anoxia by HYL-2 Ceramide Synthase. Science 2009;324:381–384. 19372430 10.1126/science.1168532

[10] Park J-W Park W-J Futerman AH Ceramide synthases as potential targets for therapeutic intervention in human diseases. Biochim Biophys Acta 2014;1841:671–681. 24021978 10.1016/j.bbalip.2013.08.019

[11] Turpin SM Nicholls HT Willmes DM Mourier A Brodesser S Wunderlich CM Mauer J Xu E Hammerschmidt P Brönneke HS Trifunovic A LoSasso G Wunderlich FT Kornfeld J-W Blüher M Krönke M Brüning JC Obesity-induced CerS6-dependent C16:0 ceramide production promotes weight gain and glucose intolerance. Cell Metab 2014;20:678–686. 25295788 10.1016/j.cmet.2014.08.002

[12] Raichur S Wang ST Chan PW Li Y Ching J Chaurasia B Dogra S Öhman MK Takeda K Sugii S Pewzner-Jung Y Futerman AH Summers SA CerS2 haploinsufficiency inhibits β-oxidation and confers susceptibility to diet-induced steatohepatitis and insulin resistance. Cell Metab 2014;20:687–695. 25295789 10.1016/j.cmet.2014.09.015

[13] Kauhanen D Sysi-Aho M Koistinen KM Laaksonen R Sinisalo J Ekroos K Development and validation of a high-throughput LC–MS/MS assay for routine measurement of molecular ceramides. Anal Bioanal Chem 2016 10.1007/s00216-016-9425-z. 26922344

[14] Granger CB Goldberg RJ Dabbous O Pieper KS Eagle KA Cannon CP Van De Werf F Avezum A Goodman SG Flather MD Fox KAA Predictors of hospital mortality in the global registry of acute coronary events. Arch Intern Med 2003;163:2345–2353. 14581255 10.1001/archinte.163.19.2345

[15] Marschner IC Colquhoun D Simes RJ Glasziou P Harris P Singh BB Friedlander D White H Thompson P Tonkin A Long-term risk stratification for survivors of acute coronary syndromes. Results from the Long-term Intervention with Pravastatin in Ischemic Disease (LIPID) Study. LIPID Study Investigators. J Am Coll Cardiol 2001;38:56–63. 11451296 10.1016/s0735-1097(01)01360-2

[16] Pencina MJ D'Agostino RB Vasan RS Evaluating the added predictive ability of a new marker: from area under the ROC curve to reclassification and beyond. Stat Med 2008;27:157–172. Discussion 207–212. 17569110 10.1002/sim.2929

[17] Battes L Akkerhuis KM Van Boven N Boersma E Kardys I Cardiovascular risk prediction models in patients with stable coronary artery disease. Exp Clin Cardiol 2014;20:117–130.

[18] Xu J Yeh CH Chen S He L Sensi SL Canzoniero LM Choi DW Hsu CY Involvement of de novo ceramide biosynthesis in tumor necrosis factor-alpha/cycloheximide-induced cerebral endothelial cell death. J Biol Chem 1998;273:16521–6. 9632721 10.1074/jbc.273.26.16521

[19] Hannun YA Functions of ceramide in coordinating cellular responses to stress. Science 1996;274:1855–1859. 8943189 10.1126/science.274.5294.1855

[20] Lozanski G Berthier F Kushner I The sphingomyelin-ceramide pathway participates in cytokine regulation of C-reactive protein and serum amyloid A, but not alpha-fibrinogen. Biochem J 1997;328 (Pt 1):271–275. 9359864 10.1042/bj3280271 PMC1218917

[21] Hamada Y Nagasaki H Fujiya A Seino Y Shang Q-L Suzuki T Hashimoto H Oiso Y Involvement of de novo ceramide synthesis in pro-inflammatory adipokine secretion and adipocyte-macrophage interaction. J Nutr Biochem 2014;25:1309–1316. 25283329 10.1016/j.jnutbio.2014.07.008

[22] Billich A Baumruker T Sphingolipid metabolizing enzymes as novel therapeutic targets. Subcell Biochem 2008;49:487–522. 18751924 10.1007/978-1-4020-8831-5_19

[23] Jänis MT Tarasov K Ta HX Suoniemi M Ekroos K Hurme R Lehtimäki T Päivä H Kleber ME März W Prat A Seidah NG Laaksonen R Beyond LDL-C lowering: distinct molecular sphingolipids are good indicators of proprotein convertase subtilisin/kexin type 9 (PCSK9) deficiency. Atherosclerosis 2013;228:380–385. 23623011 10.1016/j.atherosclerosis.2013.03.029

[24] Sachdeva A Cannon CP Deedwania PC Labresh K a Smith SC Dai D Hernandez A Fonarow GC Lipid levels in patients hospitalized with coronary artery disease: an analysis of 136 905 hospitalizations in get with the Guidelines. Am Heart J 2009;157:111–7.e2. 19081406 10.1016/j.ahj.2008.08.010

[25] Olsson AG Schwartz GG Szarek M Sasiela WJ Ezekowitz MD Ganz P Oliver MF Waters D Zeiher A High-density lipoprotein, but not low-density lipoprotein cholesterol levels influence short-term prognosis after acute coronary syndrome: results from the MIRACL trial. Eur Heart J 2005;26:890–896. 15764620 10.1093/eurheartj/ehi186

[26] Puri R Nissen SE Libby P Shao M Ballantyne CM Barter PJ Chapman MJ Erbel R Raichlen JS Uno K Kataoka Y Nicholls SJ C-reactive protein, but not low-density lipoprotein cholesterol levels, associate with coronary atheroma regression and cardiovascular events after maximally intensive statin therapy. Circulation 2013;128:2395–2403. 24043299 10.1161/CIRCULATIONAHA.113.004243

[27] Gierens H Nauck M Roth M Schinker R Schürmann C Scharnagl H Neuhaus G Wieland H März W Interleukin-6 stimulates LDL receptor gene expression via activation of sterol-responsive and Sp1 binding elements. Arterioscler Thromb Vasc Biol 2000;20:1777–1783. 10894816 10.1161/01.atv.20.7.1777

[28] Ridker PM From C-reactive protein to interleukin-6 to interleukin-1. Circ Res 2016;118:145–156. 26837745 10.1161/CIRCRESAHA.115.306656 PMC4793711

[29] Fekry B Esmaeilniakooshkghazi A Krupenko SA Krupenko NI Ceramide synthase 6 Is a novel target of methotrexate mediating its antiproliferative effect in a p53-dependent manner. PLoS ONE 2016;11:e0146618. 26783755 10.1371/journal.pone.0146618 PMC4718595

[30] Chowdhury R Khan H Heydon E Shroufi A Fahimi S Moore C Stricker B Mendis S Hofman A Mant J Franco OH Adherence to cardiovascular therapy: a meta-analysis of prevalence and clinical consequences. Eur Heart J 2013;34:2940–2948. 23907142 10.1093/eurheartj/eht295

