# Supplementary material for: Plasma ceramides predict cardiovascular death in patients with stable coronary artery disease and acute coronary syndromes beyond LDL-cholesterol
Source: Eur Heart J. 2016 Apr 28;37(25):1967–76. doi: 10.1093/eurheartj/ehw148 (PMC4929378; doi:10.1093/eurheartj/ehw148)
Supplement: Supplementary Data [file ehw148_supplementary_data.zip › ehw148supp.docx]

Plasma ceramides predict cardiovascular death in patients with stable coronary artery disease and acute coronary syndromes beyond LDL-cholesterol

Reijo Laaksonen MD ^1,2,3^, Kim Ekroos PhD ^1^, Marko Sysi-Aho PhD ^1^, Mika Hilvo PhD ^1^, Terhi Vihervaara PhD ^1^, Dimple Kauhanen BSc ^1^, Matti Suoniemi MSc ^1^, Reini Hurme PhD ^1^, Winfried März MD ^4,5^, Hubert Scharnagl PhD ^6^, Tatjana Stojakovic MD ^6^ , Efthymia Vlachopoulou MSc ^7^, Marja-Liisa Lokki PhD ^7^, Markku S. Nieminen MD ^7,18^, Roland Klingenberg MD ^8^, Christian M. Matter MD ^8^; Thorsten Hornemann MD ^9^, Peter Jüni, MD ^10^, Nicolas Rodondi, MD ^11,12^, Lorenz Räber, MD^13^, Stephan Windecker, MD ^13^, Baris Gencer ^14^, Eva Ringdal Pedersen ^15^, Grethe S Tell ^16^, Ottar Nygård * ^15,17^, Francois Mach, MD* ^14^, Juha Sinisalo MD* ^7,18^, Thomas F. Lüscher, MD* ^9^

- ^Equal contribution^

**Supplemental Appendix**

Contents

[Supplementary methods 2](#_Toc443291453)

[Study subjects 2](#_Toc443291454)

[Corogene study – all-comer CAD patients 2](#_Toc443291455)

[BECAC cohort – patients with stable CAD 2](#_Toc443291456)

[SPUM-ACS cohort – patients with ACS 3](#_Toc443291457)

[Clinical laboratory analyses 4](#_Toc443291458)

[Quantification of ceramides 4](#_Toc443291459)

[Statistical analyses 5](#_Toc443291460)

[Supplementary figures 7](#_Toc443291461)

[Supplementary tables 9](#_Toc443291462)

[References 15](#_Toc443291463)

# Supplementary methods

## Study subjects

### Corogene study – stable CAD patients

Corogene (NCT00417534) is a prospective, consecutive cohort study consisting of 5294 Finnish patients assigned to coronary angiogram in Helsinki University Central Hospital between 2006 and 2008.^1^ In order to gauge associations between high-risk ceramides and cardiovascular outcome in stable CAD patients we designed a nested case control study using the Corogene database and included data from the national death certificate registry. As cases, we selected all patients (n=80) who died from coronary heart disease within an average follow-up of 2½ years. Death registry ICD-10 codes indicative of acute myocardial infarction, atherosclerotic cardiovascular disease or ischemic heart disease (I21-I25) were used for the identification of cases. Control patients (n=80) had established CAD (>50% stenosis at least in one epicardial coronary artery), but remained alive during the follow-up period. Control patients were individually matched for age, sex, smoking, type 2 diabetes (DM2) and statin use at study entry. Baseline characteristics of the Corogene subjects are shown in Table 1 and Supplementary Table S1. Blood samples were taken at the beginning of an angiogram from arterial line and EDTA-plasma samples used for ceramide as well as other lipid analyses were stored at –80 °C until analyzed.

### BECAC cohort – patients with stable CAD

The Bergen Coronary Angiography Cohort (BECAC) includes 1580 adults referred to elective coronary angiography because of suspected stable angina pectoris. All patients were recruited at the Haukeland University Hospital, Bergen, Norway between 2000 and 2004. Information on life-style, other risk factors, previous medical history and medication was collected from questionnaires and verified against medical records. Hypertension was defined as using medical treatment for hypertension and diabetes mellitus (type 1 and 2) was defined as being previously diagnosed. Information on cardiovascular deaths was collected from the Cause of Death Registry at the Norwegian Institute of Public Health, and verified against hospital medical records whenever available. Cardiovascular disease mortality included causes of death coded I00–I99 or R96 according to the ICD-10 system. All events were adjudicated by at least two experienced physicians. Venous blood samples were drawn at baseline, usually 1-3 days before the procedure and were immediately frozen at -80 °C.

During a median follow-up of 4.6 years, a total of 81 patients died from cardiovascular disease. Baseline characteristics of the BECAC participants are reported in Table 1 and Supplementary Table S1.

### SPUM-ACS cohort – patients with ACS

Special Program University Medicine - Inflammation in Acute Coronary Syndromes (SPUM-ACS) is a prospective, multi-center (Bern, Geneva, Lausanne and Zürich) cohort study (NCT01000701). Patients with a primary diagnosis of ACS and referred for invasive management were enrolled at four Swiss university hospitals. Baseline characteristics of the SPUM-ACS patients are summarized in Table 1 and Supplementary Table S1. The study included patients of both genders, aged ≥ 18 years and presenting within five days (preferably within 72 hours) after pain onset with a main diagnosis of STEMI, NSTEMI or unstable angina. Exclusion criteria included severe physical disability, inability to comprehend the study or less than one year of life expectancy for non-cardiac reasons. Blood samples were obtained 12-24 hours after coronary angiography and plasma aliquots were stored at -80°C. Follow-up was performed at one year, with events adjudicated by independent experts. The primary endpoint was cardiac death defined as any death due to proximate cardiac cause (e.g. MI, low-output failure, fatal arrhythmia) including procedure-related deaths

SPUM-ACS, Corogene and BECAC studies were approved by the medical ethics committees in Switzerland (Cantons Zürich, Bern, Geneva and Vaud), Finland (University Central Hospital of Helsinki) and Norway (Haukeland University Hospital, Bergen), respectively, and performed in accordance with the criteria described in the declaration of Helsinki. Written informed consent was obtained from all patients.

## Clinical laboratory analyses

In Corogene subjects, total cholesterol, triacylglycerides and HDL cholesterol (homogeneous assay) were measured using enzymatic methods and reagents from DiaSys (Holzheim, Germany) and were calibrated using secondary standards from Roche Diagnostics (Mannheim, Germany) and DiaSys (for HDL cholesterol). LDL cholesterol was calculated using the Friedewald equation. ^2^ The triglyceride levels allowed the use of Friedewald equation in >99% of subjects. For SPUM and BECAC cohorts, the lipid panel parameters were similarly determined using standard methods available for each site within the study with the exception that direct LDL measurement was employed in SPUM.

In Corogene subjects, additional parameters were determined. Apolipoproteins (AI, AII, B) were measured by immunoturbidimetry using reagents from Greiner Biochemica (Flacht, Germany) and standards from Siemens (Marburg, Germany, AI, B) and from Kamiya Biomedical Company (Seattle, WA, USA, AII). Lipoprotein (a) was measured by immunoturbidimetry with reagents from Wako Diagnostics (Richmond, VA, USA) and standards from DiaSys. Lipoprotein-associated phospholipase A2 activity was measured using the PLAC® test (diaDexus Inc, San Francisco, CA, USA). The coefficients of variation (between day) were < 5%. The measurements were performed on an Olympus AU600 automatic analyzer. HDL and LDL particle numbers and sizes were measured by nuclear magnetic resonance (NMR) analysis by LipoScience, Inc. (Raleigh, North Carolina, USA) ^3^.

## Quantification of ceramides

10 µL plasma was spiked prior to extraction with deuterated internal standards; D7-Cer(d18:1/16:0), D7-Cer(d18:1/18:0), D7-Cer(d18:1/24:0) and D7-Cer(d18:1/24:1) and extracted as described in ^4^

The individual ceramides were quantified in MRM mode. Quantification was assessed through calibration line samples constructed with known amounts of synthetic Cer(d18:1/16:0), Cer(d18:1/18:0), Cer(d18:1/24:0) and Cer(d18:1/24:1) and corresponding deuterated (D7) standards. The peak area ratios of each ceramide to its corresponding deuterated form were calculated and plotted against the added ceramide concentration followed by linear regression analysis. The endogenous plasma ceramide concentrations were derived from the obtained individual regression equations by calculating corresponding concentrations from the measured peak area ratios in samples. The precision (percentage coefficient variance, % CV) and accuracy (percentage relative error, % RE) for the intra and inter for all four ceramides were within 15%. The final ceramide concentrations in plasma are presented in µM.

## Statistical analyses

Wilcoxon’s rank sum test was applied for testing differences between the *event* and *non-event* groups. Odds-ratios (OR) per standard deviation were estimated using logistic regression. ORs per 4^th^ quartile were calculated by comparing the odds between 4^th^ and 1^st^ quartiles. Hazard ratios were calculated using the Cox proportional hazard model. The GRACE^5^ risk score, consisting of Killip class, systolic blood pressure, heart rate, age, creatinine, cardiac arrest at admission, ST-segment deviation and elevated cardiac enzyme levels (troponin, CK-MB), was used to calculate the risk of long-term mortality for ACS patients. The following Marschner score^6^ variables were used in the modeling of stable CAD patient data: total cholesterol, HDL-C, age, gender, smoking status, previous acute myocardial infarction, diabetes, hypertension and prior stroke.

Validation^7^ of the c-statistics was performed as follows for the BECAC and SPUM ACS studies: in each iteration two thirds of the case subjects and two thirds of the control subjects were selected at random to fit a model that was then used to blindly predict the outcome in the remaining one third of the samples. This was repeated 1000 times and the median and the interquartile range (IQR) of the c-statistics in the blindly predicted one third of the samples were recorded. Within each iteration, the randomly selected samples used to fit and predict the outcome by the two competing models were the same (same randomization of the data applied for both model fits). The model was constructed *de novo* on each iteration. In the SPUM study, the Grace score was used as one variable in the model, and in the BECAC study the variables belonging to the Marschner score were used separately.

Net reclassification improvement (NRI) was estimated as described by Pencina et al.^8^ For the 1-year event risk of the secondary prevention population in the SPUM-ACS study we categorized subjects to low risk, intermediate risk or high-risk groups if their predicted event probability was less than 1%, 1%-5%, or more than 5%, respectively. For the BECAC study the same categorization was used for 3-year risk: only those cases who experienced CVD death within 3 years, or those controls who survived for at least 3 years were included.

The ceramide risk score was calculated as follows: For each individual all three ceramide ratios and each concentration (apart from Cer(d18:1/24:0)) were compared to the whole study population. If the variable belonged to the 3^rd^ quartile, the individual received +1 point, and if to the 4^th^ quartile, +2 points. Thus, the score ranges from 0-12 and based on the score, the subjects were split into four risk categories (0-2, 3-6, 7-9 and 10-12). When comparing the result with LDL-C, the subjects were sorted according to their LDL-C concentrations and split into four categories in the same proportion as for the ceramide risk score.

No data imputation was performed in any of the analyses. Statistical analyses were performed with R statistical software version x64 3.2.1 and SAS 9.3 software package.

# Supplementary figures

**Supplementary Figure S1:** Ceramides have been shown to mediate several key processes involved in CAD progression. Aggregated LDL within the lesion have been shown to contain 10-50 times more ceramides compared to native, unaggregated LDL^9^. Via sphingomyelinase activity, ceramide is formed at the surface of LDL particles, resulting in LDL aggregation^10^. Furthermore, ceramide participates in the transcytosis of LDL across endothelial cells^11^, followed by LDL uptake into macrophages which drives foam cell formation and vascular inflammation. Ceramides are involved in several aspects of inflammatory processes. Several cytokines, including interferon-γ, tumor necrosis factor-α (TNFα), and interleukin-1β have been shown to stimulate ceramide formation^12^. In endothelial cells ceramide has been shown to augment superoxide anion production in a process mediated by TNFα. ^13,14^ Furthermore, ceramide stimulates expression and activation of the key proinflammatory transcription factor NF-κB ^15^. Ceramides have also been implicated in endothelial dysfunction, putatively via reducing the bioavailability of nitric oxide. ^16^ Finally, ceramides are implicated in growth arrest, apoptosis and autophagy. ^17^ Medical figure elements were acquired from Servier Medical Art ([www.servier.com](http://www.servier.com)).

**Supplementary Figure S2:** The steps of ceramide synthesis are well characterized. However, it is not well understood how the arsenal of ceramides are orchestered in different biological settings. Several reaction steps have drawn special attention, as their inhibition has demonstrated potency to ameliorate pathological conditions. Inhibition of glucosylceramide synthase (GlcCer synthase), sphingomyelinase (SMase) or ceramidase is useful for treating cancer as it shifts the balance towards apoptopic ceramides. ^18^ On contrary, in inflammatory diseases the major focus has been to decrease the amount of the inflammatory ceramides, especially by inhibiting serine palmitoyltransferase (SPT) or ceramide synthases (CerS). ^19^ Regulation of CerS has attracted special attention due to the fact that the CerS family consists of six different isoforms that have different tissue distribution and fatty acyl CoA selectivity. ^20^ Getting the grip on how the various CerS can be orchestrated is pivotal, as this would immensely improve ceramide targeting by offering highly selective inhibition and activation of deleterious and protective ceramides species, respectively.

Ceramide alterations observed in the current study may derive from multiple sources. Increased ceramide synthesis in the liver (main isoforms CerS2, CerS4 and CerS5), could partially explain the increases in certain ceramides. Secondly, hepatic action of acid ceramidase was recently shown to be a significant contributor of circulating ceramides^21^, and could therefore also explain the ceramide profile alterations. Finally, the activity of SMases could be a potential source for the observed ceramide signature in the circulation, as this hydrolyzes the abundant lipoprotein-bound sphingomyelins into ceramides.


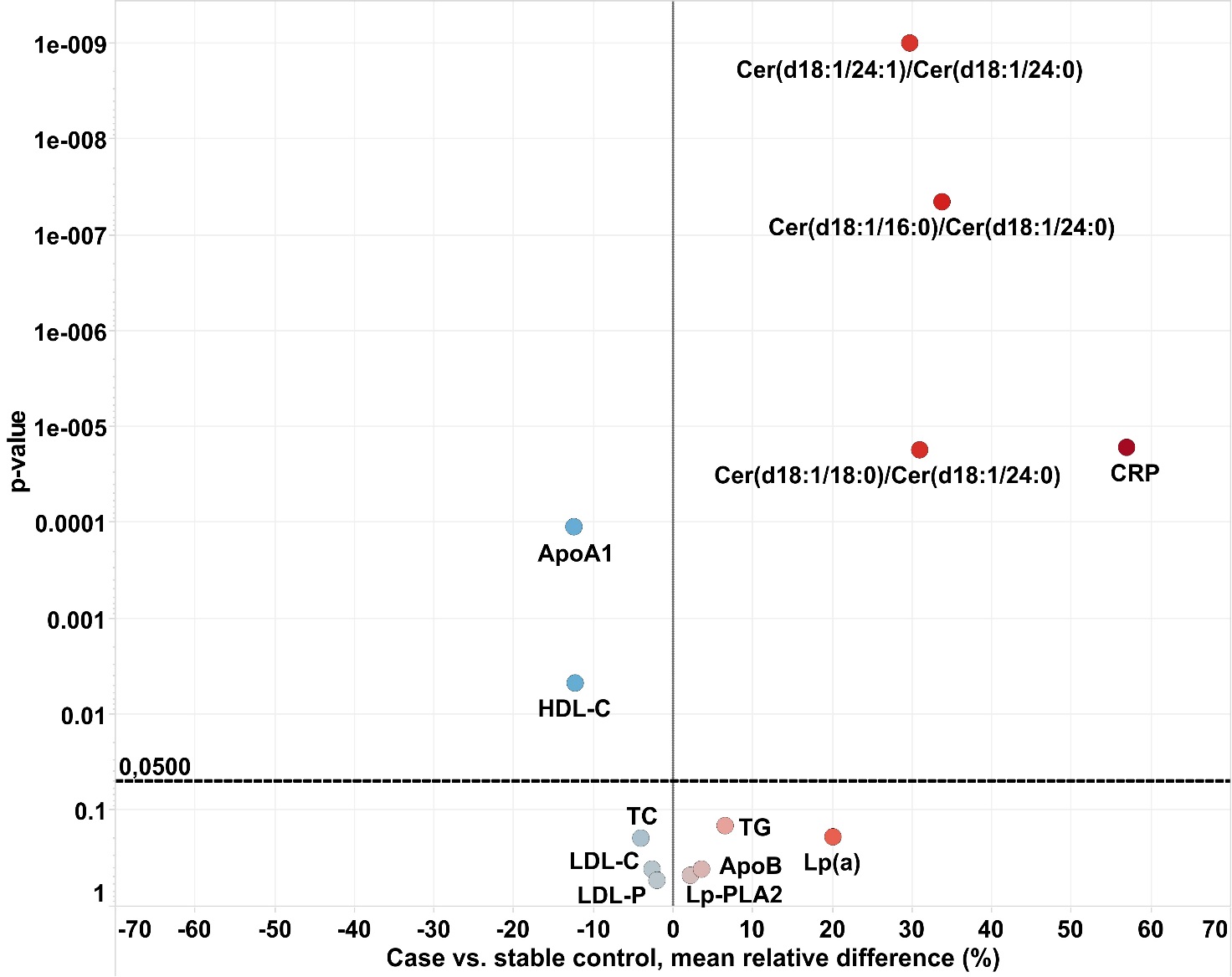


**Supplementary Figure S3:** Volcano plot illustrating the relative change and p-values of ceramides and main lipid markers in Corogene stable CAD patients.

#

**Supplementary tables**

**Supplementary Table S1:** Additional data on past medical history and medication of the patients.

|  | **COROGENE** | | **SPUM-ACS** | | **BECAC** | |
| --- | --- | --- | --- | --- | --- | --- |
| **Characteristic** | **Cases** | **Controls** | **Cases** | **Controls** | **Cases** | **Controls** |
| *No of subjects* | 80 | 80 | 51 | 1586 | 81 | 1506 |
| *Rheumatoid arthritis* |  |  |  |  |  |  |
| *Yes - no. (%)* | 4 (5%) | 2 (2%) | NA | NA | NA | NA |
| *No - no. (%)* | 76 (95%) | 78 (98%) | NA | NA | NA | NA |
| *Kidney disease* |  |  |  |  |  |  |
| *Yes - no. (%)* | 19 (24%) | 5 (6%) | NA | NA | NA | NA |
| *No - no. (%)* | 61 (76%) | 75 (94%) | NA | NA | NA | NA |
| *Tumor* |  |  |  |  |  |  |
| *Yes - no. (%)* | 7 (9%) | 9 (11%) | 12 (24%) | 108 (7%) | NA | NA |
| *No - no. (%)* | 73 (91%) | 71 (89%) | 39 (76%) | 1478 (93%) | NA | NA |
| *Beta blocker* |  |  |  |  |  |  |
| *Yes - no. (%)* | 70 (88%) | 62 (78%) | 18 (35%) | 378 (24%) | 60 (74%) | 467 (31%) |
| *No - no. (%)* | 9 (11%) | 16 (20%) | 30 (59%) | 1198 (76%) | 21 (26%) | 1039 (69%) |
| *NA* | 1 (1%) | 2 (2%) | 3 (6%) | 10 (0%) |  |  |
| *Calcium blocker* |  |  |  |  |  |  |
| *Yes - no. (%)* | 19 (24%) | 12 (15%) | 11 (22%) | 158 (10%) | 30 (37%) | 296 (20%) |
| *No - no. (%)* | 60 (75%) | 66 (82%) | 37 (73%) | 1418 (89%) | 51 (63%) | 1210 (80%) |
| *NA* | 1 (1%) | 2 (2%) | 3 (6%) | 10 (0%) |  |  |
| *ACE inhibitor* |  |  |  |  |  |  |
| *Yes - no. (%)* | 35 (44%) | 29 (36%) | 14 (27%) | 269 (17%) | 28 (35%) | 293 (19%) |
| *No - no. (%)* | 44 (55%) | 49 (61%) | 34 (67%) | 1305 (82%) | 53 (65%) | 1213 (81%) |
| *NA* | 1 (1%) | 2 (2%) | 3 (6%) | 12 (0%) |  |  |
| *AT blocker* |  |  |  |  |  |  |
| *Yes - no. (%)* | 19 (24%) | 15 (19%) | 9 (18%) | 293 (18%) | 9 (11%) | 178 (12%) |
| *No - no. (%)* | 60 (75%) | 63 (79%) | 39 (76%) | 1282 (81%) | 72 (89%) | 1328 (88%) |
| *NA* | 1 (1%) | 2 (2%) | 3 (6%) | 11 (0%) |  |  |
| *Nitrate* |  |  |  |  |  |  |
| *Yes - no. (%)* | 36 (45%) | 30 (38%) | 4 (8%) | 65 (4%) | 35 (43%) | 375 (25%) |
| *No - no. (%)* | 43 (54%) | 48 (60%) | 44 (86%) | 1515 (96%) | 46 (57%) | 1131 (75%) |
| *NA* | 1 (1%) | 2 (2%) | 3 (6%) | 6 (0%) |  |  |
| *Digitalis* |  |  |  |  |  |  |
| *Yes - no. (%)* | 15 (19%) | 2 (2%) | NA | NA | 15 (19%) | 48 (3%) |
| *No - no. (%)* | 64 (80%) | 76 (95%) | NA | NA | 66 (81%) | 1458 (97%) |
| *NA* | 1 (1%) | 2 (2%) |  |  |  |  |
| *Aspirin* |  |  |  |  |  |  |
| *Yes - no. (%)* | 54 (68%) | 63 (79%) | 17 (33%) | 486 (31%) | 58 (72%) | 1129 (75%) |
| *No - no. (%)* | 25 (31%) | 15 (19%) | 31 (61%) | 1094 (69%) | 23 (28%) | 377 (25%) |
| *NA* | 1 (1%) | 2 (2%) | 3 (6%) | 6 (0%) |  |  |
| *Insulin* |  |  |  |  |  |  |
| *Yes - no. (%)* | 25 (31%) | 11 (14%) | 7 (14%) | 79 (5%) | 4 (5%) | 52 (3%) |
| *No - no. (%)* | 54 (68%) | 67 (84%) | 41 (80%) | 1501 (95%) | 77 (95%) | 1454 (97%) |
| *NA* | 1 (1%) | 2 (2%) | 3 (6%) | 6 (0%) |  |  |
| *Diuretic* |  |  |  |  |  |  |
| *Yes - no. (%)* | 43 (54%) | 21 (26%) | 16 (31%) | 259 (16%) | 33 (41%) | 162 (11%) |
| *No - no. (%)* | 36 (45%) | 57 (71%) | 32 (63%) | 1317 (83%) | 48 (59%) | 1344 (89%) |
| *NA* | 1 (1%) | 2 (2%) | 3 (6%) | 10 (0%) |  |  |
| *Anticoagulant* |  |  |  |  |  |  |
| *Yes - no. (%)* | 18 (22%) | 10 (12%) | 6 (12%) | 50 (3%) | 16 (20%) | 82 (5%) |
| *No - no. (%)* | 61 (76%) | 68 (85%) | 42 (82%) | 1530 (96%) | 65 (80%) | 1424 (95%) |
| *NA* | 1 (1%) | 2 (2%) | 3 (6%) | 6 (0%) |  |  |
| *Clopidogrel* |  |  |  |  |  |  |
| *Yes - no. (%)* | 10 (12%) | 3 (4%) | 8 (16%) | 122 (8%) | NA | NA |
| *No - no. (%)* | 69 (86%) | 75 (94%) | 40 (78%) | 1458 (92%) | NA | NA |
| *NA* | 1 (1%) | 2 (2%) | 3 (6%) | 6 (0%) |  |  |

**Supplementary Table S2:** Medians and inter quartile ranges of established lipid markers and ceramides in case and control groups^a^. Percentages indicate the number of samples where each marker was assessed.

|  | **COROGENE** | | | | |
| --- | --- | --- | --- | --- | --- |
|  | **Cases (n=80)** | **%** | **Controls (n=80)** | **%** | **p value** |
| Cer(d18:1/16:0)/Cer(d18:1/24:0) | 0.132 (0.105-0.175) | 100 % | 0.105 (0.090-0.128) | 100 % | <0.001 |
| Cer(d18:1/18:0)/Cer(d18:1/24:0) | 0.062 (0.047-0.077) | 100 % | 0.046 (0.037-0.062) | 100 % | <0.001 |
| Cer(d18:1/24:1)/Cer(d18:1/24:0) | 0.703 (0.582-0.846) | 100 % | 0.556 (0.483-0.665) | 100 % | <0.001 |
| Cer(d18:1/16:0) (µmol/L) | 0.275 (0.222-0.326) | 100 % | 0.235 (0.212-0.282) | 100 % | 0.007 |
| Cer(d18:1/18:0) (µmol/L) | 0.118 (0.094-0.152) | 100 % | 0.107 (0.092-0.137) | 100 % | 0.195 |
| Cer(d18:1/24:0) (µmol/L) | 1.923 (1.475-2.511) | 100 % | 2.235 (1.993-2.672) | 100 % | 0.008 |
| Cer(d18:1/24:1) (µmol/L) | 1.385 (1.189-1.620) | 100 % | 1.245 (1.091-1.427) | 100 % | 0.017 |
| TC (mg/dL) | 128 (111-165) | 100 % | 139 (122-163) | 100 % | 0.064 |
| TG (mg/dL) | 108 (86-140) | 100 % | 92 (75-139) | 100 % | 0.110 |
| LDL-C (mg/dL) | 69 (55-99) | 100 % | 75 (65-92) | 100 % | 0.251 |
| LDL-P (nmol/L) | 830 (694-1110) | 93 % | 928 (712-1175) | 93 % | 0.395 |
| sdLDL (nmol/L) | 533 (304-659) | 93 % | 548 (376-737) | 93 % | 0.265 |
| ApoB (mg/dL) | 67 (55-82) | 91 % | 68.5 (57-84) | 90 % | 0.997 |
| HDL-C (mg/dL) | 34 (29-40) | 100 % | 41 (33-51) | 100 % | <0.001 |
| HDL-P (µmol/L) | 24 (21-27) | 93 % | 28 (24-31) | 93 % | <0.001 |
| sdHDL (µmol/L) | 12.8 (9.3-15.6) | 93 % | 15.8 (13.1-18.2) | 93 % | <0.001 |
| ApoA1 (mg/dL) | 115 (101-131) | 91 % | 132 (115-150) | 91 % | <0.001 |
| Lp(a) (mg/dL) | 7.2 (2-35) | 85 % | 3.6 (1-28) | 86 % | 0.319 |
| Lp-PLA2 (nmol/min/ml) | 138 (119-166) | 85 % | 130 (115-163) | 86 % | 0.354 |
| CRP (mg/L) | 3.1 (1.6-8.7) | 100 % | 1.1 (0.7-2.8) | 100 % | <0.001 |
|  | **BECAC** | | | | |
|  | **Cases (n=81)** | **%** | **Controls (n=1499)** | **%** | **p value** |
| Cer(d18:1/16:0)/Cer(d18:1/24:0) | 0.121 (0.101-0.145) | 100 % | 0.100 (0.085-0.119) | 100 % | <0.001 |
| Cer(d18:1/18:0)/Cer(d18:1/24:0) | 0.046 (0.036-0.059) | 100 % | 0.038 (0.031-0.049) | 100 % | <0.001 |
| Cer(d18:1/24:1)/Cer(d18:1/24:0) | 0.498 (0.408-0.624) | 100 % | 0.413 (0.337-0.508) | 100 % | <0.001 |
| Cer(d18:1/16:0) (µmol/L) | 0.271 (0.235-0.326) | 100 % | 0.253 (0.213-0.300) | 100 % | 0.010 |
| Cer(d18:1/18:0) (µmol/L) | 0.108 (0.077-0.143) | 100 % | 0.096 (0.076-0.123) | 100 % | 0.097 |
| Cer(d18:1/24:0) (µmol/L) | 2.335 (1.843-2.866) | 100 % | 2.548 (2.03-3.098) | 100 % | 0.035 |
| Cer(d18:1/24:1) (µmol/L) | 1.056 (0.927-1.344) | 100 % | 1.028 (0.844-1.257) | 100 % | 0.026 |
| LDL-C (mg/dL) | 110 (89-133) | 100 % | 116 (93-147) | 100 % | 0.087 |
| HDL-C (mg/dL) | 46 (35-58) | 100 % | 50 (41-62) | 100 % | 0.036 |
| TC (mg/dL) | 185 (158-212) | 100 % | 193 (166-224) | 100 % | 0.081 |
| TG (mg/dL) | 135 (100-169) | 100 % | 126 (92-182) | 100 % | 0.738 |
|  | **SPUM-ACS** | | | | |
|  | **Cases (n=51)** | **%** | **Controls (n=1586)** | **%** | **p value** |
| Cer(d18:1/16:0)/Cer(d18:1/24:0) | 0.116 (0.099-0.170) | 100 % | 0.093 (0.079-0.113) | 100 % | <0.001 |
| Cer(d18:1/18:0)/Cer(d18:1/24:0) | 0.064 (0.044-0.084) | 100 % | 0.047 (0.037-0.060) | 100 % | <0.001 |
| Cer(d18:1/24:1)/Cer(d18:1/24:0) | 0.489 (0.415-0.675) | 100 % | 0.394 (0.337-0.474) | 100 % | <0.001 |
| Cer(d18:1/16:0) (µmol/L) | 0.313 (0.255-0.385) | 100 % | 0.292 (0.247-0.346) | 100 % | 0.090 |
| Cer(d18:1/18:0) (µmol/L) | 0.161 (0.109-0.234) | 100 % | 0.146 (0.112-0.189) | 100 % | 0.163 |
| Cer(d18:1/24:0) (µmol/L) | 2.366 (2.112-3.084) | 100 % | 3.107 (2.490-3.826) | 100 % | <0.001 |
| Cer(d18:1/24:1) (µmol/L) | 1.421 (1.012-1.628) | 100 % | 1.229 (1.004-1.484) | 100 % | 0.175 |
| LDL-C (mg/dL) | 101 (81-128) | 96 % | 121 (93-150) | 97 % | 0.001 |
| HDL-C (mg/dL) | 48 (36-58) | 96 % | 44 (36-53) | 97 % | 0.266 |
| TC (mg/dL) | 159 (147-189) | 96 % | 189 (161-221) | 98 % | <0.001 |
| TG (mg/dL) | 76 (54-108) | 96 % | 92 (61-142) | 98 % | 0.014 |

^a^Cer denoted ceramide, TC total cholesterol, TG triacylglycerols, LDL-C low density lipoprotein cholesterol, HDL-C high density lipoprotein cholesterol, sdLDL small dense low density lipoprotein cholesterol, LDL-P low density lipoprotein particle number, sdHDL small dense high density lipoprotein cholesterol, HDL-P high density lipoprotein particle number, ApoB apolipoprotein B, ApoA1 apolipoprotein A1, Lp(a) lipoprotein (a), Lp-PLA2 lipoprotein-associated phospholipase A2. SI conversion factors: To convert cholesterol to mmol/L, multiply values by 0.0259; to convert triacylglycerols to mmol/L, multiply values by 0.01129.

**Supplementary Table S3:** Odds ratios per standard deviation and 4th quartile odds ratios for CV death in the SPUM ACS study.

|  | **BECAC** | | **SPUM-ACS** | |
| --- | --- | --- | --- | --- |
| **Non-adjusted** | **OR per SD** | **Q4 OR** | **OR per SD** | **Q4 OR** |
| Cer(d18:1/16:0)/Cer(d18:1/24:0) | 1.64 (1.38-1.95) | 4.84 (2.32-10.12) | 1.87 (1.55-2.24) | 14.42 (3.41-61.05) |
| Cer(d18:1/18:0)/Cer(d18:1/24:0) | 1.45 (1.21-1.75) | 3.29 (1.59-6.80) | 1.71 (1.44-2.04) | 4.76 (1.94-11.65) |
| Cer(d18:1/24:1)/Cer(d18:1/24:0) | 1.47 (1.23-1.76) | 3.40 (1.70-6.80) | 1.94 (1.59-2.36) | 5.95 (2.28-15.57) |
| Cer(d18:1/16:0) | 1.36 (1.13-1.63) | 1.99 (1.00-3.95) | 1.35 (1.08-1.69) | 1.96 (0.93-4.13) |
| Cer(d18:1/18:0) | 1.25 (1.05-1.50) | 1.57 (0.87-2.85) | 1.36 (1.08-1.72) | 1.23 (0.60-2.52) |
| Cer(d18:1/24:0) | 0.82 (0.64-1.04) | 0.67 (0.35-1.26) | 0.46 (0.32-0.67) | 0.18 (0.07-0.48) |
| Cer(d18:1/24:1) | 1.35 (1.12-1.61) | 2.36 (1.15-4.87) | 1.16 (0.90-1.49) | 1.74 (0.86-3.50) |
| TC | 0.88 (0.70-1.11) | 0.66 (0.35-1.25) | 0.57 (0.41-0.79) | 0.20 (0.08-0.54) |
| TG | 1.03 (0.83-1.27) | 0.75 (0.37-1.52) | 0.53 (0.31-0.93) | 0.35 (0.14-0.89) |
| LDL-C | 0.93 (0.74-1.17) | 0.51 (0.25-1.03) | 0.61 (0.45-0.83) | 0.23 (0.09-0.61) |
| HDL-C | 0.83 (0.65-1.06) | 0.50 (0.25-1.00) | 1.14 (0.87-1.50) | 1.37 (0.66-2.87) |
| **Adjusted ^*^** |  |  |  |  |
| Cer(d18:1/16:0)/Cer(d18:1/24:0) | 1.65 (1.38-1.98) | 5.06 (2.37-10.80) | 1.64 (1.29-2.08) | 6.79 (1.48-31.21) |
| Cer(d18:1/18:0)/Cer(d18:1/24:0) | 1.43 (1.18-1.74) | 3.16 (1.49-6.73) | 1.49 (1.17-1.90) | 3.11 (1.07-9.05) |
| Cer(d18:1/24:1)/Cer(d18:1/24:0) | 1.47 (1.23-1.76) | 3.50 (1.71-7.16) | 1.68 (1.29-2.19) | 2.95 (1.00-8.71) |
| Cer(d18:1/16:0) | 1.99 (1.52-2.61) | 3.48 (1.51-8.01) | 1.71 (1.26-2.32) | 3.42 (1.27-9.22) |
| Cer(d18:1/18:0) | 1.43 (1.13-1.82) | 1.84 (0.91-3.70) | 1.51 (1.10-2.06) | 1.93 (0.74-5.08) |
| Cer(d18:1/24:0) | 0.75 (0.54-1.04) | 0.59 (0.27-1.32) | 0.68 (0.40-1.16) | 0.44 (0.12-1.64) |
| Cer(d18:1/24:1) | 1.70 (1.33-2.17) | 3.11 (1.39-6.94) | 1.62 (1.14-2.31) | 3.76 (1.47-9.59) |

***^*^****Adjusted for TC, TG, HDL-C, LDL-C and CRP.*

*Cer denoted ceramide, TC total cholesterol, TG triacylglycerols, LDL-C low density lipoprotein cholesterol and HDL-C high density lipoprotein cholesterol*

**Supplementary Table S4:** Odds ratios in BECAC and SPUM ACS cohorts with and without statin adjustment in the models^a^.

|  | **BECAC** | | | |
| --- | --- | --- | --- | --- |
|  | **OR per SD** | | **Q4 OR** | |
|  | **Statin adjustment-** | **Statin adjustment+** | **Statin adjustment-** | **Statin adjustment+** |
| Cer(d18:1/16:0)/Cer(d18:1/24:0) | 1.65 (1.38-1.98) | 1.68 (1.40-2.01) | 5.06 (2.37-10.80) | 5.15 (2.41-11.05) |
| Cer(d18:1/18:0)/Cer(d18:1/24:0) | 1.43 (1.18-1.74) | 1.42 (1.16-1.73) | 3.16 (1.49-6.73) | 2.95 (1.38-6.30) |
| Cer(d18:1/24:1)/Cer(d18:1/24:0) | 1.47 (1.23-1.76) | 1.48 (1.23-1.77) | 3.50 (1.71-7.16) | 3.42 (1.67-7.02) |
| Cer(d18:1/16:0) | 1.99 (1.52-2.61) | 1.97 (1.51-2.59) | 3.48 (1.51-8.01) | 3.56 (1.54-8.24) |
| Cer(d18:1/18:0) | 1.43 (1.13-1.82) | 1.40 (1.10-1.79) | 1.84 (0.91-3.70) | 1.71 (0.84-3.45) |
| Cer(d18:1/24:0) | 0.75 (0.54-1.04) | 0.74 (0.53-1.03) | 0.59 (0.27-1.32) | 0.60 (0.27-1.32) |
| Cer(d18:1/24:1) | 1.70 (1.33-2.17) | 1.68 (1.31-2.15) | 3.11 (1.39-6.94) | 3.02 (1.35-6.76) |
|  |  |  |  |  |
|  | **SPUM ACS** | | | |
|  | **OR per SD** | | **Q4 OR** | |
|  | **Statin adjustment-** | **Statin adjustment+** | **Statin adjustment-** | **Statin adjustment+** |
| Cer(d18:1/16:0)/Cer(d18:1/24:0) | 1.64 (1.29-2.08) | 1.63 (1.28-2.08) | 6.79 (1.48-31.21) | 6.68 (1.45-30.83) |
| Cer(d18:1/18:0)/Cer(d18:1/24:0) | 1.49 (1.17-1.90) | 1.47 (1.14-1.88) | 3.11 (1.07-9.05) | 2.77 (0.94-8.21) |
| Cer(d18:1/24:1)/Cer(d18:1/24:0) | 1.68 (1.29-2.19) | 1.63 (1.24-2.14) | 2.95 (1.00-8.71) | 2.63 (0.88-7.88) |
| Cer(d18:1/16:0) | 1.71 (1.26-2.32) | 1.65 (1.20-2.25) | 3.42 (1.27-9.22) | 2.97 (1.09-8.11) |
| Cer(d18:1/18:0) | 1.51 (1.10-2.06) | 1.44 (1.04-1.99) | 1.93 (0.74-5.08) | 1.47 (0.54-4.03) |
| Cer(d18:1/24:0) | 0.68 (0.40-1.16) | 0.65 (0.38-1.11) | 0.44 (0.12-1.64) | 0.44 (0.12-1.67) |
| Cer(d18:1/24:1) | 1.62 (1.14-2.31) | 1.49 (1.04-2.16) | 3.76 (1.47-9.59) | 3.23 (1.25-8.36) |

*^a^The models were adjusted for TC, TG, HDL-C, LDL-C, CRP as well as with and without statin treatment*

**Supplementary Table S5:** Odds ratios in BECAC cohort in patients that were on or not on statin treatment both at baseline and after 1-year follow-up.

| **WITHOUT STATIN** | **OR per SD** | **OR 4th Quartile** |
| --- | --- | --- |
| Cer(d18:1/16:0)/Cer(d18:1/24:0) | 1.81 (1.31-2.49) | NA^c^ |
| Cer(d18:1/16:0) | 1.64 (1.13-2.37) | 4.25 (0.88-20.45) |
| Cer(d18:1/18:0) | 1.49 (1.07-2.07) | 1.66 (0.53-5.22) |
| Cer(d18:1/24:0) | 0.59 (0.34-1.00) | 0.24 (0.05-1.15) |
| Cer(d18:1/24:1) | 1.87 (1.28-2.73) | 2.81 (0.73-10.87) |
| LDL-C | 1.03 (0.65-1.65) | 0.73 (0.20-2.66) |
|  |  |  |
| **WITH STATIN** | **OR per SD** | **OR 4th Quartile** |
| Cer(d18:1/16:0)/Cer(d18:1/24:0) | 1.59 (1.28-1.98) | 3.68 (1.64-8.29) |
| Cer(d18:1/16:0) | 1.34 (1.09-1.65) | 2.64 (1.14-6.12) |
| Cer(d18:1/18:0) | 1.17 (0.94-1.46) | 1.36 (0.68-2.73) |
| Cer(d18:1/24:0) | 0.95 (0.73-1.24) | 0.79 (0.40-1.56) |
| Cer(d18:1/24:1) | 1.27 (1.03-1.57) | 2.79 (1.07-7.26) |
| LDL-C | 1.11 (0.87-1.41) | 1.06 (0.52-2.14) |

*^a^n=18 cases, 460 controls*

*^b^n=61 cases, 879 controls*

*^c^1st quartile did not have any cases, thus calculation cannot be performed*

**Supplementary Table S6:** Multivariable model for SPUM ACS study.

|  | **SPUM** | |
| --- | --- | --- |
|  | **Multivariable 3** | |
|  | **Hazard ratio (95% CI)** | **p value** |
| Cer(d18:1/16:0)/Cer(d18:1/24:0) | 1.77 (1.36-2.31) | p<0.001 |
| Cer(d18:1/18:0)/Cer(d18:1/24:0) | 1.68 (1.32-2.13) | p<0.001 |
| Cer(d18:1/24:1)/Cer(d18:1/24:0) | 1.69 (1.29-2.21) | p<0.001 |
| Cer (d18:1/16:0) | 2.31 (1.54-3.46) | p<0.001 |
| Cer (d18:1/18:0) | 2.26 (1.54-3.30) | p<0.001 |
| Cer (d18:1/24:0) | 0.90 (0.57-1.43) | 0.667 |
| Cer (d18:1/24:1) | 2.02 (1.32-3.09) | 0.001 |

***^a^****adjusted with HDL-C, LDL-C, triglycerides, age, BMI, systolic blood pressure, diabetes status, smoking and stratified for gender*

**Supplementary Table S7:** Hazard ratios for non-fatal Q-wave and nonQ-wave myocardial infarctions in the SPUM study.

|  | **SPUM Q-wave (15 events)** | | **SPUM nonQ-wave (30 events)** | |
| --- | --- | --- | --- | --- |
|  | **Multivariable** | | **Multivariable** | |
|  | **Hazard ratio (95% CI)** | **p value** | **Hazard ratio (95% CI)** | **p value** |
| Cer(d18:1/16:0)/Cer(d18:1/24:0) | 1.78 (1.25-2.53) | 0.001 | 1.21 (0.88-1.67) | 0.243 |
| Cer(d18:1/18:0)/Cer(d18:1/24:0) | 1.74 (1.24-2.44) | 0.001 | 1.02 (0.72-1.44) | 0.926 |
| Cer(d18:1/24:1)/Cer(d18:1/24:0) | 1.53 (0.95-2.47) | 0.083 | 1.30 (0.95-1.77) | 0.106 |
| Cer (d18:1/16:0) | 1.58 (0.88-2.85) | 0.127 | 1.27 (0.84-1.90) | 0.255 |
| Cer (d18:1/18:0) | 1.63 (0.95-2.82) | 0.078 | 1.07 (0.73-1.57) | 0.742 |
| Cer (d18:1/24:0) | 0.67 (0.34-1.30) | 0.236 | 0.95 (0.59-1.51) | 0.814 |
| Cer (d18:1/24:1) | 1.07 (0.59-1.97) | 0.818 | 1.37 (0.89-2.10) | 0.155 |

**Supplementary Table S8:** Correlation of distinct ceramides with plasma LDL-C and CRP^a^

|  | **COROGENE** | | **SPUM-ACS** | |
| --- | --- | --- | --- | --- |
|  | **LDL-C** | **CRP** | **LDL-C** | **CRP** |
| **All patients** |  |  |  |  |
| Cer(d18:1/16:0) | 0.53*** | 0.28*** | 0.37*** | 0.19*** |
| Cer(d18:1/18:0) | 0.24** | 0.39*** | 0.27*** | 0.34*** |
| Cer(d18:1/24:0) | 0.62*** | -0.12 | 0.57*** | -0.15*** |
| Cer(d18:1/24:1) | 0.49*** | 0.16* | 0.34*** | 0.14*** |
| **Cases only** |  |  |  |  |
| Cer(d18:1/16:0) | 0.55*** | 0.16 | 0.27 | 0.32*** |
| Cer(d18:1/18:0) | 0.32** | 0.21 | 0.24 | 0.40** |
| Cer(d18:1/24:0) | 0.63*** | -0.17 | 0.67*** | -0.27 |
| Cer(d18:1/24:1) | 0.46*** | -0.02 | 0.36* | 0.13 |
| **Controls only** |  |  |  |  |
| Cer(d18:1/16:0) | 0.58*** | 0.26* | 0.38*** | 0.18*** |
| Cer(d18:1/18:0) | 0.19 | 0.54*** | 0.27*** | 0.34*** |
| Cer(d18:1/24:0) | 0.56*** | 0.04 | 0.56*** | -0.13*** |
| Cer(d18:1/24:1) | 0.60*** | 0.21 | 0.34*** | 0.14*** |

*^a^Values are Spearman’s correlation coefficients, * p<0.05, ** p<0.01, *** p<0.001*

**Supplementary Table S9:** Correlation of distinct ceramide ratios with plasma LDL-C and CRP^a^

| **Study** | **variable** | **Cer(d18:1/16:0)/Cer(d18:1/24:0)** | **Cer(d18:1/18:0)/Cer(d18:1/24:0)** | **Cer(d18:1/24:1)/Cer(d18:1/24:0)** |
| --- | --- | --- | --- | --- |
| Corogene | LDL-C | -0.19* | -0.28*** | -0.24** |
| SPUM-ACS | LDL-C | -0.31*** | -0.21*** | -0.32*** |
|  |  |  |  |  |
| Corogene | CRP | 0.32*** | 0.45*** | 0.35*** |
| SPUM-ACS | CRP | 0.35*** | 0.47*** | 0.34*** |

*^a^Values are Spearman’s correlation coefficients, * p<0.05, ** p<0.01, *** p<0.001*

**Supplementary Table S10:** CRP and Cer(d18:1/16:0)/Cer(d18:1/24:0) quartiles in the BECAC and SPUM studies. Margins show the result when the population is categorized only by one variable.

| **BECAC** | | **Cer(d18:1/16:0)/Cer(d18:1/24:0)** | | | | |
| --- | --- | --- | --- | --- | --- | --- |
|  |  | **q1** | **q2** | **q3** | **q4** | **margin** |
| **CRP** | **q1** | 1.0 % | 3.9 % | 3.8 % | 5.4 % | *3.5 %* |
|  | **q2** | 1.1 % | 2.8 % | 1.9 % | 12.0 % | *4.1 %* |
|  | **q3** | 2.5 % | 1.1 % | 4.1 % | 11.1 % | *4.5 %* |
|  | **q4** | 4.5 % | 3.3 % | 12.5 % | 11.5 % | *8.3 %* |
|  | **margin** | *2.3 %* | *2.8 %* | *5.3 %* | *10.1 %* |  |
| **SPUM** | | **Cer(d18:1/16:0)/Cer(d18:1/24:0)** | | | | |
|  |  | **q1** | **q2** | **q3** | **q4** | **margin** |
| **CRP** | **q1** | 0.0 % | 0.9 % | 3.7 % | 0.0 % | *1.1 %* |
|  | **q2** | 0.0 % | 1.0 % | 0.0 % | 1.8 % | *0.5 %* |
|  | **q3** | 1.0 % | 4.1 % | 3.1 % | 2.5 % | *2.7 %* |
|  | **q4** | 2.9 % | 4.8 % | 3.2 % | 11.4 % | *7.3 %* |
|  | **margin** | *0.5 %* | *2.4 %* | *2.5 %* | *6.4 %* |  |

**Supplementary Table S11:** Calculation of ceramide risk score.

| **Score component** | **3rd quartile** | **4th quartile** |
| --- | --- | --- |
| Cer(d18:1/16:0)/Cer(d18:1/24:0) | +1 | +2 |
| Cer(d18:1/18:0)/Cer(d18:1/24:0) | +1 | +2 |
| Cer(d18:1/24:1)/Cer(d18:1/24:0) | +1 | +2 |
| Cer(d18:1/16:0) | +1 | +2 |
| Cer(d18:1/18:0) | +1 | +2 |
| Cer(d18:1/24:1) | +1 | +2 |

# References

1 Vaara S, Nieminen MS, Lokki M-L, Perola M, Pussinen PJ, Allonen J, Parkkonen O, Sinisalo J. Cohort Profile: The Corogene study. *Int J Epidemiol* 2012; **41**: 1265–71.

2 Friedewald WT, Levy RI, Fredrickson DS. Estimationof the Concentration of Low-Density Lipoprotein Cholesterolin Plasma,Without Use of the Preparative Ultracentrifuge. *Clin Chem* 1972; **18**: 499–502.

3 Otvos JD, Mora S, Shalaurova I, Greenland P, Mackey RH, Goff DC. Clinical implications of discordance between low-density lipoprotein cholesterol and particle number. *J Clin Lipidol*; **5**: 105–13.

4 Kauhanen D, Sysi-Aho M, Koistinen KM, Laaksonen R, Sinisalo J, Ekroos K. Development and validation of a high-throughput LC–MS/MS assay for routine measurement of molecular ceramides. *Anal Bioanal Chem* 2016. DOI:10.1007/s00216-016-9425-z.

5 Granger CB, Goldberg RJ, Dabbous O, Pieper KS, Eagle KA, Cannon CP, Van De Werf F, Avezum A, Goodman SG, Flather MD, Fox KAA. Predictors of hospital mortality in the global registry of acute coronary events. *Arch Intern Med* 2003; **163**: 2345–53.

6 Marschner IC, Colquhoun D, Simes RJ, Glasziou P, Harris P, Singh BB, Friedlander D, White H, Thompson P, Tonkin A. Long-term risk stratification for survivors of acute coronary syndromes. Results from the Long-term Intervention with Pravastatin in Ischemic Disease (LIPID) Study. LIPID Study Investigators. *J Am Coll Cardiol* 2001; **38**: 56–63.

7 Kohavi R. A Study of Cross-Validation and Bootstrap for Accuracy Estimation and Model Selection. In: International Joint Conference on Artificial Intelligence. 1995: 1137–43.

8 Pencina MJ, D’Agostino RB, Vasan RS. Evaluating the added predictive ability of a new marker: from area under the ROC curve to reclassification and beyond. *Stat Med* 2008; **27**: 157–72; discussion 207–12.

9 Schissel SL, Tweedie-Hardman J, Rapp JH, Graham G, Williams KJ, Tabas I. Rabbit aorta and human atherosclerotic lesions hydrolyze the sphingomyelin of retained low-density lipoprotein. Proposed role for arterial-wall sphingomyelinase in subendothelial retention and aggregation of atherogenic lipoproteins. *J Clin Invest* 1996; **98**: 1455–64.

10 Walters MJ, Wrenn SP. Effect of Sphingomyelinase-mediated generation of ceramide on aggregation of low-density lipoprotein. *Langmuir* 2008; **24**: 9642–7.

11 Li W, Yang X, Xing S, Bian F, Yao W, Bai X, Zheng T, Wu G, Jin S. Endogenous Ceramide Contributes to the Transcytosis of oxLDL across Endothelial Cells and Promotes Its Subendothelial Retention in Vascular Wall. *Oxid Med Cell Longev* 2014; **2014**: 1–11.

12 Marathe S. Human Vascular Endothelial Cells Are a Rich and Regulatable Source of Secretory Sphingomyelinase. IMPLICATIONS FOR EARLY ATHEROGENESIS AND CERAMIDE-MEDIATED CELL SIGNALING. *J Biol Chem* 1998; **273**: 4081–8.

13 Xu J, Yeh CH, Chen S, He L, Sensi SL, Canzoniero LM, Choi DW, Hsu CY. Involvement of de novo ceramide biosynthesis in tumor necrosis factor-alpha/cycloheximide-induced cerebral endothelial cell death. *J Biol Chem* 1998; **273**: 16521–6.

14 Modur V, Zimmerman GA, Prescott SM, McIntyre TM. Endothelial cell inflammatory responses to tumor necrosis factor alpha. Ceramide-dependent and -independent mitogen-activated protein kinase cascades. *J Biol Chem* 1996; **271**: 13094–102.

15 Nixon GF. Sphingolipids in inflammation: Pathological implications and potential therapeutic targets. *Br J Pharmacol* 2009; **158**: 982–93.

16 Zhang DX, Zou A-P, Li P-L. Ceramide Reduces Endothelium-Dependent Vasodilation by Increasing Superoxide Production in Small Bovine Coronary Arteries. *Circ Res* 2001; **88**: 824–31.

17 Gomez-Muñoz A, Presa N, Gomez-Larrauri A, Rivera I-G, Trueba M, Ordoñez M. Control of inflammatory responses by ceramide, sphingosine 1-phosphate and ceramide 1-phosphate. *Prog Lipid Res* 2016; **61**: 51–62.

18 Morad SAF, Cabot MC. Ceramide-orchestrated signalling in cancer cells. *Nat Rev Cancer* 2013; **13**: 51–65.

19 Schmitz-Peiffer C. Targeting ceramide synthesis to reverse insulin resistance. *Diabetes* 2010; **59**: 2351–3.

20 Levy M, Futerman AH. Mammalian Ceramide Synthases. *IUBMB Life* 2011; **62**: 347–56.

21 Xia JY, Holland WL, Kusminski CM, Sun K, Sharma AX, Pearson MJ, Sifuentes AJ, McDonald JG, Gordillo R, Scherer PE. Targeted Induction of Ceramide Degradation Leads to Improved Systemic Metabolism and Reduced Hepatic Steatosis. *Cell Metab* 2015; **22**: 266–78.
